# Integrated analysis of multimodal single-cell data

**DOI:** 10.1016/j.cell.2021.04.048

**Published:** 2021-06-24

**Authors:** Yuhan Hao, Stephanie Hao, Erica Andersen-Nissen, William M. Mauck, Shiwei Zheng, Andrew Butler, Maddie J. Lee, Aaron J. Wilk, Charlotte Darby, Michael Zager, Paul Hoffman, Marlon Stoeckius, Efthymia Papalexi, Eleni P. Mimitou, Jaison Jain, Avi Srivastava, Tim Stuart, Lamar M. Fleming, Bertrand Yeung, Angela J. Rogers, Juliana M. McElrath, Catherine A. Blish, Raphael Gottardo, Peter Smibert, Rahul Satija

**Affiliations:** 1 Center for Genomics and Systems Biology, New York University, New York, NY 10003, USA; 2 New York Genome Center, New York, NY 10013, USA; 3 Technology Innovation Lab, New York Genome Center, New York, NY 10013, USA; 4 Vaccine and Infectious Disease Division, Fred Hutchinson Cancer Research Center, Seattle, WA 98109, USA; 5 Cape Town HVTN Immunology Lab, Hutchinson Cancer Research Institute of South Africa, Cape Town 8001, South Africa; 6 Department of Medicine, Stanford University School of Medicine, Stanford, CA 94305, USA; 7 Center for Data Visualization, Fred Hutchinson Cancer Research Center, Seattle, WA 98109, USA; 8 BioLegend Inc., San Diego, CA 92121, USA; 9 Chan Zuckerberg Biohub, San Francisco, CA 94063, USA

**Keywords:** single cell genomics, multimodal analysis, CITE-seq, immune system, T cell, reference mapping, COVID-19

## Abstract

The simultaneous measurement of multiple modalities represents an exciting frontier for single-cell genomics and necessitates computational methods that can define cellular states based on multimodal data. Here, we introduce “weighted-nearest neighbor” analysis, an unsupervised framework to learn the relative utility of each data type in each cell, enabling an integrative analysis of multiple modalities. We apply our procedure to a CITE-seq dataset of 211,000 human peripheral blood mononuclear cells (PBMCs) with panels extending to 228 antibodies to construct a multimodal reference atlas of the circulating immune system. Multimodal analysis substantially improves our ability to resolve cell states, allowing us to identify and validate previously unreported lymphoid subpopulations. Moreover, we demonstrate how to leverage this reference to rapidly map new datasets and to interpret immune responses to vaccination and coronavirus disease 2019 (COVID-19). Our approach represents a broadly applicable strategy to analyze single-cell multimodal datasets and to look beyond the transcriptome toward a unified and multimodal definition of cellular identity.

## Highlights

• “Weighted nearest neighbor” analysis integrates multimodal single-cell data
• A multimodal reference “atlas” of the circulating human immune system
• Identification and validation of novel sources of lymphoid heterogeneity
• “Reference-based” mapping of query datasets onto a multimodal atlas

## Graphical abstract

**Figure:**
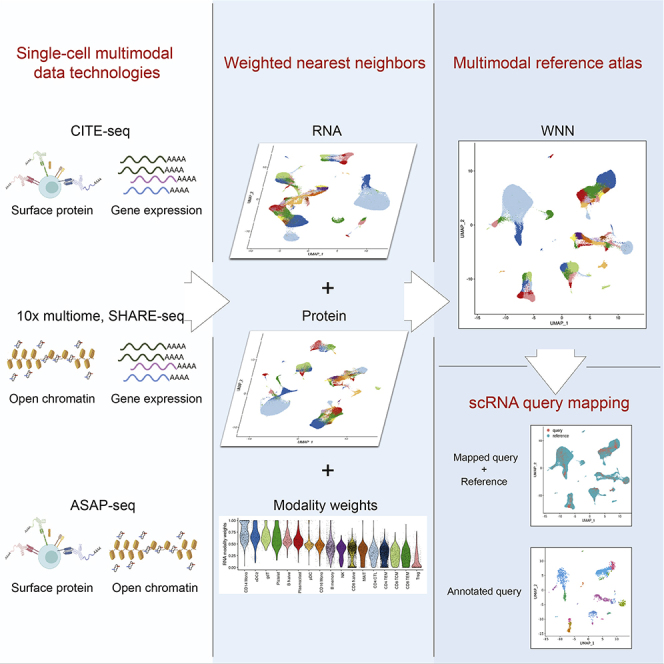

## Teaser

A framework that allows for the integration of multiple data types using single cells is applied to understand distinct immune cell states, previously unidentified immune populations, and to interpret immune responses to vaccinations.

## Introduction

The potential to catalog and characterize the rich diversity of cell types in the human immune system represents a powerful opportunity for single-cell genomics (Chen et al., 2019a; Gomes et al., 2019; Jaitin et al., 2014; Papalexi and Satija, 2018; Stubbington et al., 2017), yet also reveals the limitations of current approaches. Although established technologies like single-cell RNA-seq (scRNA-seq) are capable of discovering new cell types and states in heterogeneous tissues, transcriptomics alone is often incapable of separating molecularly similar, but functionally distinct, categories of immune cells. Despite tremendous functional diversity, distinct populations of T cells such as effector, regulatory, γδ, and mucosal associated invariant T (MAIT), often cannot be effectively separated by scRNA-seq alone, even when using the most sensitive and cutting-edge technologies (Ding et al., 2020; Mereu et al., 2020). This reflects technical challenges driven by the minimal RNA content of T cells coupled with high RNase expression (Andreeff et al., 1978; Lu et al., 2018; Sercan Alp et al., 2015), which hampers scRNA-seq data quality. More broadly, this exhibits the challenge of defining cell states based on the transcriptome alone, because important sources of cellular heterogeneity may not correlate strongly with transcriptomic features despite being identifiable in other modalities.

Multimodal single-cell technologies, which simultaneously profile multiple data types in the same cell, represent a new frontier for the discovery and characterization of cell states. For example, we recently introduced CITE-seq (Stoeckius et al., 2017), which leverages oligonucleotide-conjugated antibodies to simultaneously quantify RNA and surface protein abundance in single cells via the sequencing of antibody-derived tags (ADTs). Moreover, pioneering technological advancements now enable the simultaneous profiling of transcriptome alongside either chromatin accessibility (Cao et al., 2018; Chen et al., 2019b), DNA methylation (Gaiti et al., 2019; Luo et al., 2019), nucleosome occupancy (Clark et al., 2018; Pott, 2017), or spatial location (Rodriques et al., 2019; Vickovic et al., 2019). Each of these approaches offers an exciting solution to overcome the inherent limitations of scRNA-seq and to explore how multiple cellular modalities affect cellular state and function (Zhu et al., 2020).

The maturation of multimodal single-cell technologies also necessitates the development of new computational methods to integrate information across different data types (Efremova and Teichmann, 2020). For example, although CITE-seq datasets can be analyzed by first identifying clusters based on gene expression values (Peterson et al., 2017; Stoeckius et al., 2017) and subsequently exploring their immunophenotypes, a multimodal computational workflow would define cell states based on both modalities. Importantly, these strategies must be robust to potentially large differences in the data quality and information content for each modality. In some contexts, robust protein quantifications may be most valuable for clustering, especially with a large and well-designed antibody panel. In other contexts (particularly when important cell type markers are missing or not previously known), the unsupervised nature of a cell’s transcriptome may be the most valuable. The varying information content of each modality, even across cells in the same dataset, represents a pressing challenge for the analysis and integration of multimodal datasets.

Here, we introduce “weighted-nearest neighbor” (WNN) analysis, an analytical framework to integrate multiple data types measured within a cell and to obtain a joint definition of cellular state. Our approach is based on an unsupervised strategy to learn cell-specific modality “weights,” which reflect the information content for each modality and determine its relative importance in downstream analyses. We demonstrate that WNN analysis substantially improves our ability to define cellular states in multiple biological contexts and data types. We leverage this method to generate a multimodal “atlas” based on a CITE-seq dataset of 211,000 human peripheral blood mononuclear cells (PBMCs), with large cell-surface protein marker panels extending up to 228 antibodies. We utilize this dataset to identify and validate heterogeneous cell states in human lymphocytes and explore how the human immune system responds to vaccination and severe acute respiratory syndrome coronavirus 2 (SARS-CoV-2) infection. Our approach, implemented in an updated version 4 of our open source R toolkit Seurat, represents a broadly applicable strategy for integrative multimodal analysis of single-cell data.

## Results

### Quantifying the relative utility of each modality in each cell

We sought to design a robust analytical workflow for the integration of multiple measurements collected within the same cell. To be applied to a range of biological contexts and data types, our strategy must successfully address the following criteria. First, the workflow must be robust to potentially vast differences in data quality between the modalities. Second, integrative multimodal analysis should enable multiple downstream analytical tasks, including visualization, clustering, and the identification of cellular trajectories. Last, and most importantly, simultaneous analysis of multiple modalities should improve on the ability to discover and characterize cell states, compared to independent analyses of each modality when performed separately.

These challenges highlight the importance of a flexible framework to handle diverse datasets. As previously described for CITE-seq (Mimitou et al., 2019; Stoeckius et al., 2017), the increased copy number of protein molecules compared to RNA molecules typically leads to more robust detection of protein features. The protein data in CITE-seq may therefore represent the most informative modality, particularly in cases where the antibody panel comprehensively represents all cell subsets with high specificity. Other panels may omit antibodies for key or previously undiscovered markers, or contain antibodies with low binding specificity, in which case the unsupervised nature of scRNA-seq may be most informative. Even within the same dataset, the relative utility of each modality to define cell states may vary across individual cells.

**Figure 1:**
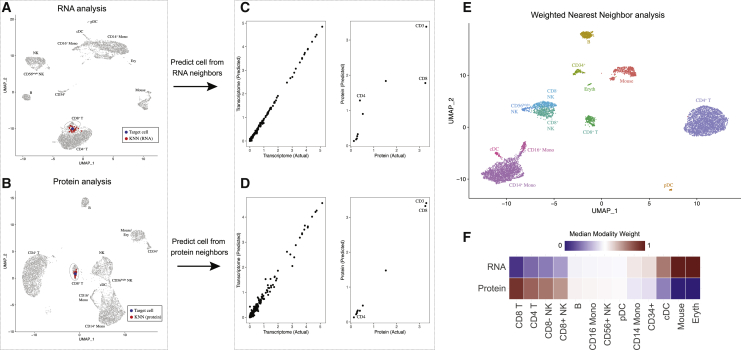
Schematic overview of multimodal integration using weighted nearest neighbor analysis (A and B) Independent analysis of transcriptome (A) and protein (B) modalities from a CITE-seq dataset of cord blood mononuclear cells. Blue dot marks the same target cell in (A) and (B). Red dots denote the k = 20 nearest neighbors to the target cell based on the transcriptome (A) or protein (B) modalities. (C) The RNA neighbors are averaged together to predict the molecular contents of the target cell, which can be compared to the actual measurements. Each dot denotes an individual gene, and the axis scale of expression is based on default log-normalization in Seurat. Because the RNA neighbors represent a mixture of different T cell subsets, there is substantial error between predicted and measured protein expression levels for CD4 and CD8. (D) Same as in (C), but averaging protein neighbors. Because protein neighbors are all CD8 T cells, the predicted values are close to the actual measurements. We can therefore infer that for this target cell, the protein data are most useful for defining cell state and assign it a higher protein modality weight. As described in STAR Methods, we perform the prediction and comparison steps in low-dimensional space. (E) We can integrate the modalities by constructing a weighted nearest neighbor (WNN) graph, based on a weighted average of protein and RNA similarities. UMAP visualization and clustering of this graph. (F) Median RNA and protein modality weights for all cell types in the dataset. Modality weights were calculated for each cell without knowledge of cell type labels. See also Figure S1.

**Figure S1:**
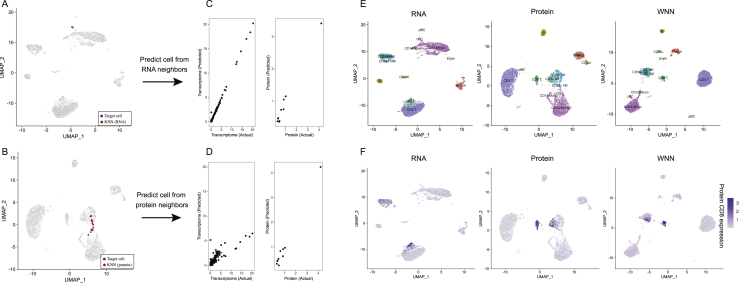
Weighted nearest neighbor analysis on a CITE-seq dataset of cord blood mononuclear cells, related to Figure 1 (A, B) Independent analysis of transcriptome (A) and protein (B) modalities from a CITE-seq analysis of cord blood mononuclear cells. Panels A-D correspond to Figures 1A–1D, but the target cell is a dendritic cell instead of a CD8 T cell. Blue dot marks the same target dendritic cell in (A) and (B). Red dots denote the k = 20 nearest neighbors to the target dendritic cell based on the transcriptome (A) or protein (B) modalities. (C) The RNA neighbors are averaged together to predict the molecular contents of the target dendritic cells. Since the RNA neighbors are all dendritic cells, the predicted values are close to the actual measurements. (D) Same as in (C), but averaging protein neighbors. Since protein neighbors are a mixture of cell types, there is substantial error between predicted and measured RNA expression. Thus, the RNA data is more informative for characterizing the state of the target cell, and the cell is assigned an increased RNA modality weight. (E) RNA, Protein and WNN UMAP visualization for this dataset. Cells are annotated by their WNN-assigned labels. Visualizations are the same as in Figure 1, but all cell types are labeled on the UMAP plots for greater clarity. (F) Feature plot of CD8 protein expression on all three UMAP visualizations, showing that WNN and ADT analyses help to separate CD4 and CD8 T cells, and also identify additional heterogeneity within NK cells.

We therefore designed an analytical solution to address these goals, without requiring prior knowledge from the user regarding the importance of each modality. We first introduce and demonstrate our solution on our previously generated CITE-seq dataset of 8,617 cord blood mononuclear cells, with a panel of 10 immunophenotypic markers (Stoeckius et al., 2017). Independent unsupervised analysis of the RNA and protein data revealed largely consistent cell classifications (Figures 1A, 1B, and S1) but did exhibit some differences. For example, CD8^+^ and CD4^+^ T cells were partially blended together when analyzing the transcriptome but separated clearly in the protein data. Contrastingly, conventional dendritic cells (cDCs), along with a rare population of erythroid progenitors and spiked-in murine 3T3 controls, formed distinct clusters when analyzing RNA but were intermixed with other cell types based on surface protein abundance. With biological foresight, the cell-type-specific differences across modalities could be predicted by the composition of the CITE-seq panel, which contained anti-CD4 and anti-CD8 antibodies but lacked any immunophenotypic markers to discriminate cDCs.

For each cell, we began by independently calculating sets of *k* = 20 nearest neighbors for each modality. We found that for CD8^+^ T cells, the most similar RNA neighbors often reflected a mix of CD8^+^ and CD4^+^ T cells (in the RNA KNN graph, there are a total of 944 incorrect edges that connect CD8^+^ to CD4^+^ T cells). By contrast, protein neighbors were predominantly correctly identified as CD8^+^ T cells (in the protein KNN graph, 12 CD8^+^/CD4^+^ edges were identified). This reflects the particular utility of protein data when defining the state of these cells. Next, we independently averaged the molecular profiles of protein neighbors and RNA neighbors (i.e., predicted the molecular contents of a cell from its neighbors), and compared the averages to their original measured values. We found that for CD8^+^ T cells, protein KNN-based predictions were more accurate compared to RNA KNN-based predictions (Figures 1C and 1D), whereas the converse was true for cDCs (Figure S1).

We then leveraged the relative accuracy of these predictions to calculate RNA and protein modality “weights,” describing their relative information content for each individual cell. We provide a detailed mathematical description for each component of the WNN workflow in the STAR Methods, highlighting three key steps: (1) obtaining within modality and cross-modality predictions, (2) converting these to prediction affinities, based on a cell-specific bandwidth kernel, and (3) calculating modality weights using a softmax transformation. The RNA and protein modality weights are non-negative, unique to each cell, and sum to 1.

Our final step integrates the modalities to create a WNN graph. For each cell, we calculate a new set of *k*-nearest cells based on a metric that reflects the weighted average of normalized RNA and protein similarities (STAR Methods). The WNN graph is a single representation of a multimodal dataset, but should more accurately reflect the richness of both data types. For example, the WNN graph contained only 20 CD8^+^/CD4^+^ edges. Moreover, many common analytical tasks for single-cell data—including t-distributed stochastic neighbor embedding)/uniform manifold approximation and projection (t-SNE/UMAP) visualization, clustering, and trajectory inference—can accept a user-specified neighbor graph as input. We therefore used our WNN graph to derive an integrated UMAP and clustering of our CITE-seq dataset (Figure 1E). In contrast to the separate analysis of either modality, our joint integration clearly separated CD4^+^ and CD8^+^ T cells, retained the identity of cDCs, and also uncovered additional sources of subtle heterogeneity within natural killer (NK) cells (Figure S1). We observed that cells classified as CD8^+^ T cells were assigned higher protein modality weights, whereas DCs were assigned higher RNA modality weights, recapitulating our biological expectations despite the fact that the calculation of modality weights was unsupervised and unaware of cell-type labels (Figure 1F).

### WNN analysis is a robust and flexible approach for multimodal analysis

We next further explored the performance of our WNN integration, assessed its robustness to fluctuations in data quality, and performed benchmarking against other recently developed methods. For these analyses, we used a more recently generated CITE-seq dataset of human bone marrow, representing 30,672 mononuclear cells with a panel of 25 antibodies. While the samples contained cells across the full spectrum of hematopoietic differentiation, the antibody panel was designed to separate groups of terminally differentiated cells.

**Figure 2:**
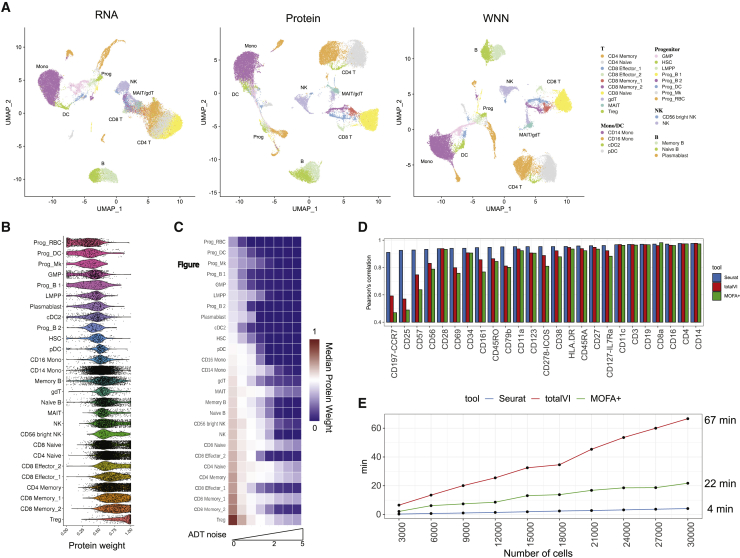
Benchmarking and robustness analysis for WNN integration (A) Analysis of a CITE-seq dataset of human bone marrow mononuclear cells and 25 surface proteins. UMAP visualizations are computed using RNA, protein, or WNN analysis. Cell annotations are derived from WNN analysis and reveal heterogeneity within T cells and progenitors that cannot be discovered by either modality independently. Granular annotations, which more clearly indicate subpar performance when analyzing only one modality, are shown in Figure S2. (B) Single-cell protein modality weights. Progenitor populations all receive low protein weights, whereas T cell populations receive high protein modality weights, consistent with the composition of the antibody panel that was tailored for differentiated cell types. (C) To test the robustness of WNN, we added increasing amounts of Gaussian noise to the protein data. Protein weights decrease to 0 in all cell types as noise levels increase. (D and E) Benchmarking WNN against totalVI and MOFA+. (D) The integrated latent space defined by WNN most accurately reconstructs expression levels for 25 proteins. (E) WNN analysis exhibits improved runtimes compared to competing methods. Additional benchmarking analyses in Figure S2. See also Figure S3.

**Figure S2:**
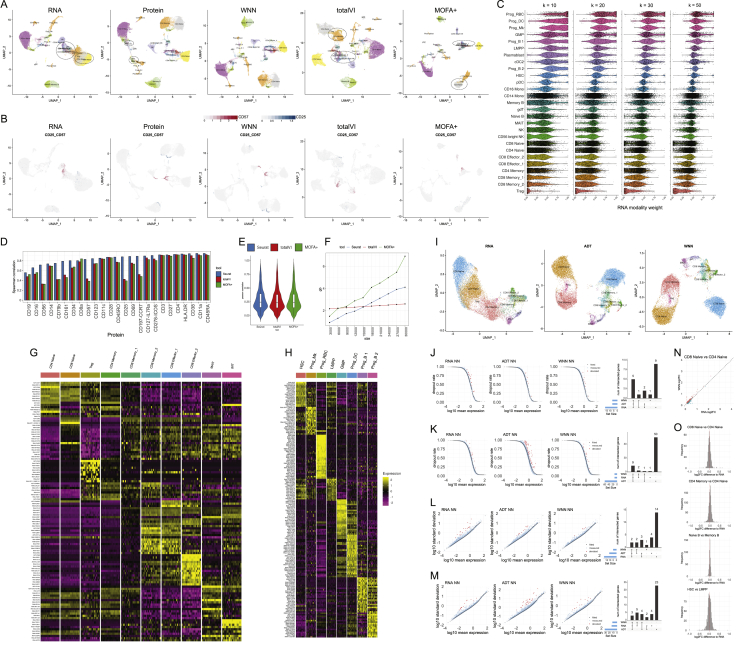
Benchmarking and robustness analysis for WNN integration on a CITE-seq dataset of human bone marrow mononuclear cells (BMNC), related to Figure 2 (A) UMAP visualizations of the BMNC dataset based on five analytical strategies: independent RNA analysis, independent Protein analysis, WNN, totalVI and MOFA+. Cell annotations are derived from WNN analysis, which reflect distinct molecular states (see heatmaps in (G-H)). Dashed ovals indicate regions in each analysis where cell states are intermixed. (B) Expression of protein CD25 and CD57 in these five UMAP visualizations. In WNN analysis, cells that are positive for these proteins are correctly determined to be neighbors of each other, and therefore separate in UMAP visualization. (C) Robustness analysis for k in the WNN procedure (k = 20 by default). We varied the number of single-cell RNA modality weights across different number of k-nearest neighbors used (k = 10, 20, 30, 50) on the BMNC dataset, and show single-cell violin plots of the resulting RNA modality weight. We observe only minor fluctuations when varying k within this range. (D) Benchmarking WNN against totalVI and MOFA+. The integrated latent space defined by WNN most accurately reconstructs expression levels for all 25 proteins. Same as Figure 2D but showing Spearman correlation instead of Pearson correlation. (E) When using the integrated latent space to reconstruct 2000 variable features in the transcriptome, all three methods exhibit equivalent performance. Figure shows boxplot of Pearson correlation between predicted and measured values for 2,000 features. Benchmarking metrics are described further in STAR Methods. (F) Memory usage for all three methods as a function of the size of the input dataset. (G) Heatmap of WNN-annotated T cell states. Features include the best RNA and protein features identified by differential expression. Heatmap displays pseudobulk averages where cells are grouped by cell type, human donor, and technical replicate, and demonstrates that markers are repeatedly detected across samples and replicates. (H) Same as in (G) but for progenitor cell states. (I) Sub-clustering BMNC T cells based on RNA profiles, ADT profiles, and WNN analysis. (J) Gene dropout curve for neighbors of regulatory T cells defined by RNA, ADT, and WNN analysis. Each point represents a gene, with the average trendline in black. Genes that deviate from the trendline (STAR Methods) are denoted as ‘variable’ and plotted as red dots. Rightmost panel represents an upsetR plot examining the set of variable genes identified for each neighborhood set, and shows that WNN-derived neighborhoods exhibit a lower number of variable genes than RNA-derived neighborhoods. (K) Same as in (J) but for HSC cells. (L) Same as (J) but examining the standard deviation of gene expression as an alternative metric to dropout rate. (M) Same as in (L) but for HSC cells. (N) Absolute log2FC of differentially expressed genes between CD4 Naive and CD8 Naive clusters, where clusters were defined by either RNA or WNN analysis (STAR Methods). (O) Distribution of changes in the magnitude of log2FC for differentially expressed genes between cell populations based on WNN-based and RNA-based clustering. Distributions are centered at 0, indicating that for all comparisons, WNN-derived clusters were equally effective at identifying cluster-enriched genes as RNA-derived clusters.

Consistent with our previous example, WNN integration substantially increased our ability to resolve hematopoietic cell states (Figures 2A and S2). Once cell states were annotated through integrated multimodal clustering, we were able to discover differentially expressed (DE) genes and proteins in each group, further validating their biological identity and significance (Figure S2). However, although these cell types are defined by both RNA and protein markers, the statistical power in unsupervised analysis of either modality separately was insufficient to identify these populations, demonstrating the importance of joint analysis. Indeed, when examining the cell-specific modality weights, we found that T cell groups—and in particular, populations that were masked in scRNA-seq analyses—all received higher protein modality weights (Figure 2B). We found that unsupervised transcriptome-focused clustering was unable to separate these cell states, even if we performed a focused re-clustering using only T cells (Figure S2).

Conversely, each of the cell populations with the highest RNA weights represented hematopoietic progenitor populations. As a result, our multimodal analysis was able to identify diverse populations of hematopoietic stem cells, lymphoid-primed multipotent progenitors (LMPP), and progenitors of erythroid, platelet, monocyte, B, and conventional/plasmacytoid DC lineages that could be recovered in scRNA-seq data, even though these groups lacked immunophenotypic markers in our CITE-seq experiment. We confirmed that our results were robust to a range of values for *k* (Figure S2), and the incorporation of protein information in the WNN graph does not come at the expense of identifying transcriptomically congruent neighborhoods (Figure S2; STAR Methods).

These results suggest that integrated WNN analysis can provide necessary flexibility and allow one data type to compensate for weaknesses in another. We confirmed this using a simulation experiment, where we added increasing amounts of random Gaussian noise to the ADT data, in order to mimic increases in nonspecific binding (Figure 2C). We found that the increasing ADT noise led to a decrease in protein weights for all cell types, in a dose-dependent manner. Moreover, protein modality weights were assigned to 0 after a sufficient amount of protein noise was added, correctly instructing downstream analyses to focus only on scRNA-seq data.

We next benchmarked WNN analysis against two recently introduced methods for multimodal integration: multi-omics factor analysis v2 (MOFA+) (Argelaguet et al., 2020), which uses a statistical framework based on factor analysis, and totalVI (Gayoso et al., 2019), which combines deep neural networks with a hierarchical Bayesian model. Both methods integrate the modalities into a latent space, which we used to construct an integrated *k*-NN graph and a 2D UMAP visualization. We reasoned that we could quantify the performance of the different methods by comparing the similarity of each cell’s molecular state to its closest neighbors in the integrated latent space. We found that for each of the 25 proteins (Figure 2D), as well as the RNA transcriptome (Figure S2), WNN analysis exhibited superior or equivalent performance to alternative approaches. The difference in performance was particularly striking for markers of regulatory (CD25) and effector (CD57) T cells. This was consistent with UMAP visualization, in which WNN was the only method where these populations were not intermixed with other groups (Figure S2). WNN analysis also exhibited significant improvements in speed, ranging up to 15-fold when analyzing the full dataset (Figure 2E).

**Figure S3:**
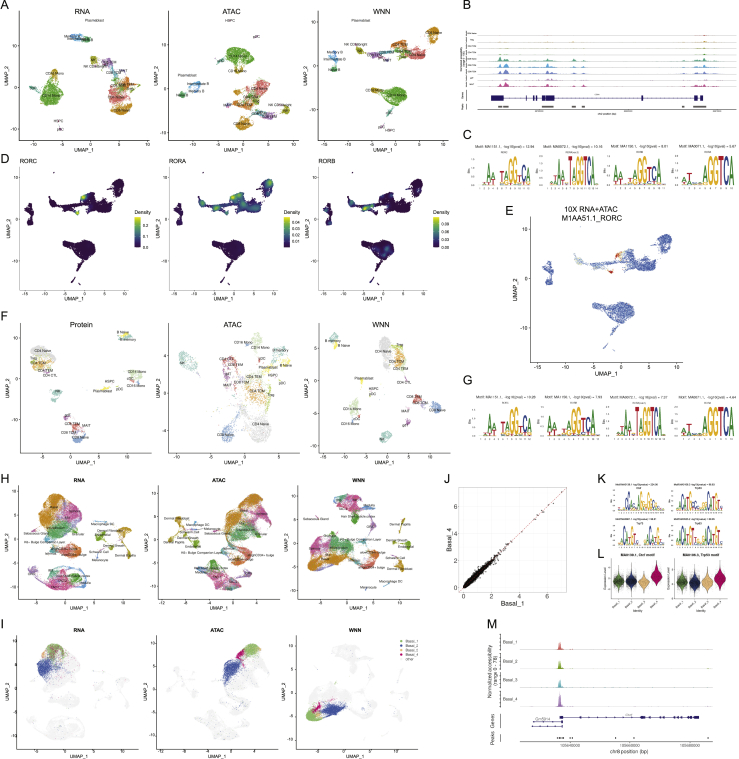
Applying WNN to additional multimodal technologies, related to Figure 2 (A) Analysis of a publicly available dataset of 11,351 PBMC processed with the 10x Genomics Multiome ATAC+RNA kit. UMAP visualizations of RNA and ATAC-seq data, as well as integrated WNN analysis. Cells are labeled by their WNN-annotated clusters. (B) Visualization of pseudobulk chromatin accessibility tracks of the CD8A locus for eight T cell subsets. Multiple peaks clearly separate CD8+ and CD8- T cells, exemplifying the information in ATAC-seq that can enhance parallel RNA measurements for defining cell states. (C) Enriched motifs within MAIT-specific open chromatin regions. Since multiple transcription factors (i.e., RORA, RORB, RORC) have very similar binding motifs, each exhibits strong evidence of enrichment. (D) Density plots, produced by the Nebulosa package, showing the RNA expression of RORC, RORA and RORB. (E) Visualization of RORC motif activity, as calculated by chromVAR, which mirrors the expression of the RORC as shown in (D). (F) Analysis of a published ASAP-seq dataset of 4,725 human PBMC where chromatin accessibility and surface expression of 227 surface proteins are simultaneously measured. UMAP visualizations of ATAC and protein data, as well as integrated WNN analysis. Cells are labeled by their WNN-annotated clusters. (G) Enriched motifs within MAIT-specific open chromatin regions in the ASAP-seq dataset are concordant with those identified in ATAC+RNA analysis. (H) Analysis of a publicly available dataset of 34,774 mouse skin cells from SHARE-seq, which generates paired single-cell profiles of gene expression and chromatin accessibility. UMAP visualizations of RNA and ATAC-seq profiles, as well as integrated WNN analysis. Cells are labeled by their annotations from (Ma et al., 2020b). (I) Four basal subpopulations were identified from WNN clustering, and cells from each subpopulation are highlighted in the UMAP visualizations from (H). Basal_4 and Basal_1 do not separate in transcriptomic analysis, but form distinct clusters in ATAC and WNN analysis. (J) Pseudobulk expression profiles of the Basal_4 and Basal_1 subpopulations demonstrate that the two groups exhibit similar transcriptomic profiles. (K) Top motifs exhibiting differential accessibility between Basal_4 and Basal_1, as identified by chromVar analysis. (L) chromVar motif activity scores for the p53 and CTCF motifs for all basal subpopulations. In each case, Basal_4 exhibits elevated accessibility at these motif sites. ^∗∗∗^p value < 1e-5 based on Wilcoxon test. (M) Visualization of pseudobulk chromatin accessibility tracks of the Ct*cf.* locus for four basal subpopulations. In addition to exhibiting greater accessibility globally at CTCF motif sites, Basal_4 exhibits increased accessibility at the Ct*cf.* promoter.

Although we primarily demonstrate our approach on CITE-seq datasets, our strategy is applicable to diverse multimodal technologies. For example, recent developments have enabled the simultaneous measurement of ATAC-seq profiles and transcriptomes from single nuclei (Cao et al., 2018; Chen et al., 2019b). We applied WNN analysis to a dataset of 11,351 paired PBMC profiles generated by the 10x Genomics Multiome ATAC+RNA kit. We found that the combination of modalities exhibited maximal power to separate immune subsets (Figure S3). Interestingly, similar to our CITE-seq analyses, we found that ATAC-seq data were more capable of separating naive CD8^+^ and CD4^+^ T cell states due to reliable detection of cell-type-specific open chromatin regions (Figure S3). The separation of these clusters upon UMAP visualization (Figure S3) was consistent with the number of incorrect naive CD8^+^/CD4^+^ edges identified in each representation (RNA KNN: 984, ATAC KNN: 373, WNN: 322).

The combination of ATAC and RNA data also allowed us to identify differentially accessible DNA sequence motifs between our WNN-defined clusters. For example, we found that ATAC-seq peaks accessible in MAIT cells were highly enriched for motifs for the pro-inflammatory transcription factor RORγt (Ivanov et al., 2006; Willing et al., 2018), which was also upregulated transcriptionally in these cells (Figure S3). We obtained highly concordant results when applying WNN analysis to ASAP-seq (Mimitou et al., 2020), a third multimodal technology, that pairs measurements of surface protein abundance with ATAC-seq profiles in single cells (Figure S3).

Last, we considered a recent dataset of 34,774 mouse skin cells generated by SHARE-seq (Ma et al., 2020), which generates paired measurements of chromatin accessibility and gene expression. WNN analysis recapitulated each of the 23 populations described in the original manuscript where unsupervised clustering was performed on transcriptomic measurements, including three subgroups of Basal cells that could be distinguished from scRNA-seq. However, in addition to the published findings, WNN analysis identified a novel population of Basal cells that exhibits distinct chromatin accessibility profiles, but does not exhibit unique transcriptomic characteristics (Figure S3). As basal cells in the skin are continually replenished (Epstein, 2008), cells that exhibit a primed chromatin state preceding transcriptomic shifts may differ in their proliferative and regenerative potential. We found that the Basal_4 population was specifically characterized by increased chromatin accessibility at CTCF and p53 motifs (Demirkan et al., 2000) (Figure S3). Notably, basal cell carcinoma, the most common form of skin cancer, is often characterized by mutations in p53 and CTCF binding sites (Poulos et al., 2016) and results in uncontrolled basal cell division. Taken together, these findings demonstrate that the ability of WNN to identify subpopulations that are masked by scRNA-seq alone is not limited to immune or CITE-seq datasets. We conclude that WNN analysis is capable of sensitively and robustly characterizing populations that cannot be identified by a single modality, exhibits best-in-class performance, and can be flexibly applied to multiple data types for integrative and multimodal analysis.

### A multimodal atlas of the human PBMCs

Although flow cytometry and cytometry by time of flight (CyTOF) are widely used and powerful approaches for making high-dimensional measurements of protein expression in immune cells (Bendall et al., 2011; Bodenmiller et al., 2012; Diggins et al., 2015; Saeys et al., 2016), CITE-seq’s use of distinct oligonucleotide barcode sequences provides a unique opportunity to profile very large panels of antibodies alongside cellular transcriptomes. In addition, we have recently demonstrated that the signal-to-noise for each antibody can be optimized for any individual marker as a function of antibody concentration, and we have shown that CITE-seq data quality does not deteriorate with greater amounts of total antibody (Stoeckius et al., 2018). We therefore curated and optimized a panel of TotalSeqA reagents encompassing 228 antibodies (Table S1) comprising a diverse set of lineage and activation markers.

**Figure 3:**
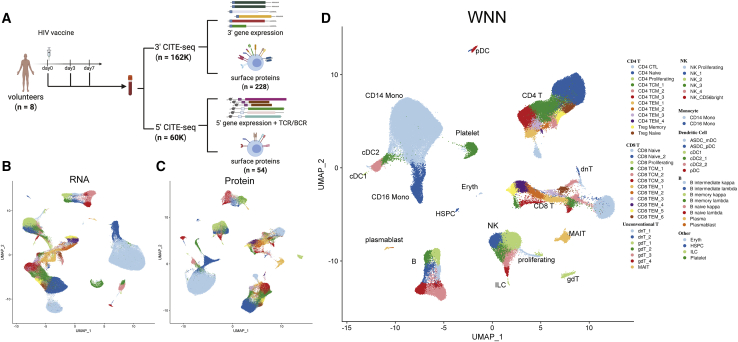
A multimodal atlas of human PBMC (A) Experimental design schematic of the CITE-seq experiment. PBMC samples originate from eight volunteers pre (day 0) and post-vaccination (day 3 and day 7). We processed each sample with CITE-seq using the 10x 3′ (228 antibodies) and 10x 5′ (54 antibodies + BCR + TCR) technologies, yielding a total of 210,911 cells. (B–D) UMAP visualization of 161,764 cells 10x 3′ cells analyzed based on RNA data (B), protein data (C), or WNN analysis (D). Cell types were identified using unsupervised clustering of the WNN graph and grouped into three annotation tiers, ranging from eight broad categories, to 57 high-resolution clusters. UMAP visualization of 49,147 10x 5′ cells, mapped onto the 3′ reference data, is shown in Figure S5. See also Table S1.

We leveraged the CITE-seq technology alongside our optimized antibody panel and integrative WNN analysis strategy to generate a multimodal atlas of human PBMCs. We obtained PBMC samples from eight volunteers enrolled in an HIV vaccine trial (Elizaga et al., 2018; Li et al., 2017), with ages spanning from 20–49 years. For each subject, PBMCs were collected at three time points: immediately before (day 0), 3 days, and 7 days following administration of a VSV-vectored HIV vaccine (Figure 3A). For each sample, we profiled cells using 10x Chromium 3′ (using 228 TotalSeq A antibodies), representing a total of 161,764 cells (average of 8,003 unique RNA molecules/cell, 5,251 unique ADT/cell). We also profiled a total of 49,147 cells (54 antibodies) split across all samples using ECCITE-seq (Mimitou et al., 2019), which also enables immune repertoire profiling with the 10x 5′ technology. After NovaSeq sequencing, stringent quality control, and doublet filtration (STAR Methods), our final dataset consists of 210,911 total cells and allows us to profile cellular heterogeneity in both the resting (unvaccinated) and activated (post-vaccination) immune system.

We applied our “anchor-based” workflow (Stuart et al., 2019) to first integrate the samples together, enabling cells to cluster together based on their shared biological state, as opposed to sample-of-origin (STAR Methods). Although this causes unvaccinated and vaccinated samples to cluster together initially, it enables us to annotate cell states consistently in all samples, and to learn cell-type-specific responses in downstream analyses. We then performed joint analysis of both modalities using WNN integration, and as a comparative control, visualized the dataset using the RNA and protein modalities independently (Figures 3B–3D).

**Figure S4:**
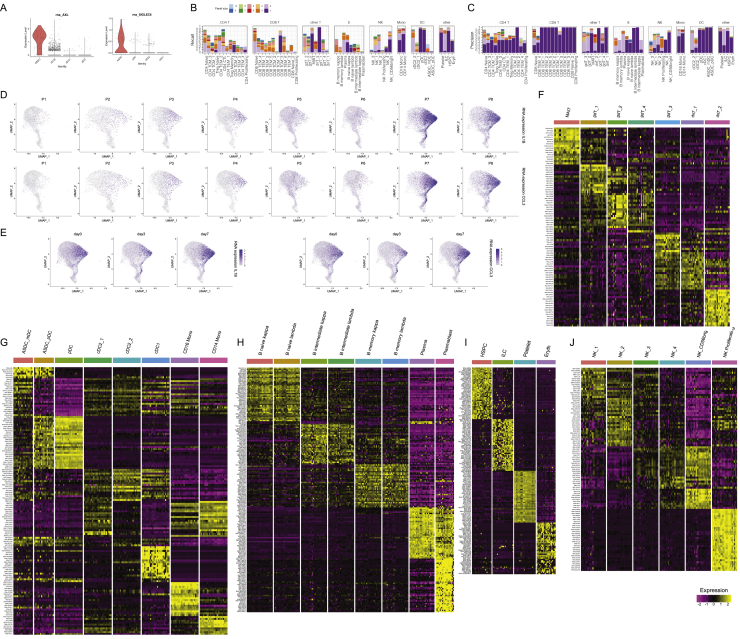
Identifying targeted gene expression markers and immunophenotype panels, related to Figure 4 (A) RNA expression of two canonical markers of AXL+ SIGLEC6+ dendritic cells (ASDC). Both markers were specifically enriched in the ASDC cells compared to other DC subsets. (B-C) For each of the 57 clusters, we computed targeted immunophenotype panels using forward selection coupled with logistic regression. In Figure 4C we visualize the level of enrichment for each cluster based on panels of one to ten markers. Here, we show precision and recall metrics based on logistic regression, using a decision boundary of 0.5. These data demonstrate that while we can achieve substantial enrichment with small panels, isolating pure and homogeneous populations based on small marker panels remains challenging for some clusters. (D-E) Additional heterogeneity in the expression of inflammatory genes in monocyte populations. Only CD14+ and CD16+ monocytes are shown. Heterogeneous expression of these genes is exhibited in multiple, but not all, volunteers. This heterogeneity was not related to the vaccination time course, as shown in (E). (F) Heatmap of unconventional T cells states. Features include the best RNA and protein features identified by differential expression. Heatmap displays pseudobulk averages where cells are grouped by cell type, human volunteer, and vaccination time point and demonstrates that markers are repeatedly identified across samples. Heatmaps for CD4+ T cell and CD8+ T cell states are shown in Figures 4A and 4B. (G) Same as in (F) but for myeloid cell states. (H) Same as in (F) but for B cell states. B cell states are subdivided by their mutually exclusive expression of kappa or lambda light chain, with distinguishing markers including IGKC, IGLC3, IGLC3. (I) Same as in (F) but for other cells states. (J) Same as in (F) but for NK cells states.

We identified 57 clusters in WNN analysis, encapsulating all major and minor immune cell types and revealing striking cellular diversity particularly within lymphoid lineages. With rare exceptions for infrequent cell types, each cluster included cells from all 24 samples. Our clusters could be readily grouped into larger categories, including CD4^+^ T cells (12 clusters), CD8^+^ T cells (12 clusters), unconventional T cells (7 clusters), NK cells (6 clusters), B cells, plasma cells, and plasmablasts (8 clusters), dendritic cells and monocytes (8 clusters), and rare clusters of hematopoietic progenitors, platelets, erythrocytes, and circulating innate lymphoid cells (ILC). To assist in the interpretation of our clusters, we assign each cell three annotations with increasing granularity (level 1, 8 categories; level 2, 30 categories; level 3, 57 categories). Although we saw the greatest level of heterogeneity within T cell subsets, our analysis clearly identified heterogeneous subsets of myeloid cells that were fully concordant with recent high-resolution scRNA-seq analyses of sorted populations, including extremely rare populations (0.02%) of dendritic cells defined by the expression of *AXL* and *SIGLEC6* (See et al., 2017; Villani et al., 2017) (ASDC; Figure S4). We also identified substantial heterogeneity in the expression of inflammatory genes such as *IL1B* and *CCL3* within monocyte populations, but because this heterogeneity varied across different volunteers, we conservatively did not further subdivide these states (Figure S4).

**Figure 4:**
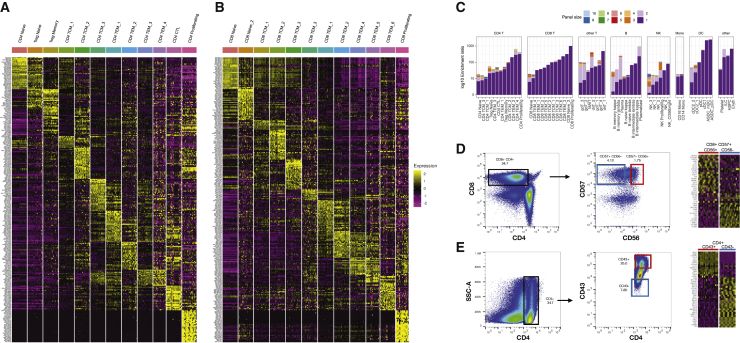
Multimodal biomarkers of immune cell states (A) Heatmap of CD4^+^ T cell states. Markers include the best RNA and protein features identified by differential expression (DE). Heatmap displays pseudobulk averages where cells are grouped by cell type, donor, and vaccination time point and demonstrates that markers do not vary across different PBMC samples. (B) Same as in (A) but for CD8^+^ T cell states. Additional heatmaps are shown in Figure S4. (C) For each of our 57 clusters, we calculated the optimal surface marker enrichment panels based on our CITE-seq data. Bar plots show the ability of the panels to enrich for each cell type *in silico*. The composition of each panel is shown in Table S2. (D) Validation of predicted marker panels for the CD8_TEM_5 cluster. We sorted cells based on the marker panels identified in (C), and performed bulk RNA-seq. Each column represents a replicate bulk RNA-seq profile. Heatmap is ordered by genes expected to be DE based on our CITE-seq dataset and are validated by bulk RNA-seq. (E) Same as in (D) but for CD4 CTL cells.

We next identified differentially expressed RNA and immunophenotype markers for each cluster. We found that each cluster exhibited distinct molecular patterns and biomarkers for both modalities (Figure 4A; additional heatmaps in Figure S4). Moreover, these identified biomarkers were invariant across human volunteers and vaccination time points. Despite the fact that clusters were enriched for both RNA and protein markers, our ability to identify these groups was substantially reduced without WNN analysis, as multiple clusters blended together when performing separate analysis of either RNA or protein data (Figures 3B and 3C). We conclude that multimodal integration is essential for the unsupervised discovery and annotation of immune cell states; however, once these states are enumerated, supervised differential analyses are capable of sensitively describing markers that define their molecular state.

Due to the robust detection of protein features in CITE-seq combined with the size of our antibody panel, we reasoned that we could discover small panels of immunophenotypic markers to perform targeted enrichment through flow cytometry. We used stepwise variable selection coupled with logistic regression (STAR Methods) to identify the best antibody marker panels of different sizes (1–10 markers) for each subset, and calculated the level of enrichment *in silico* (Figure 4C). We found that a single marker was capable of achieving effective enrichment of at least 10-fold for 45 clusters, whereas a panel with three markers was sufficient to achieve 10-fold enrichment for 55 clusters.

We confirmed that this marker discovery procedure identifies effective panels for well-characterized populations (plasmacytoid DC [pDC]: CD123^+^; MAIT cells: CD3^+^ CD161^+^ TCRvɑ7.2^+^; CD4 naive: CD4^+^ CD45RA^+^ CD45RB^+^). In other instances (e.g., cytotoxic populations of CD4^+^ lymphocytes), our analysis identified CD43 as a marker with high enrichment power that has not been previously reported. For this population, as well as a subgroup of highly cytotoxic CD8^+^ T cells (CD8_TEM_5), we successfully validated our enrichment panels in an independent set of PBMCs from healthy donors by conventional flow cytometry followed by bulk RNA-seq (STAR Methods). In both cases, we examined the expression level of genes that we expected to be DE-based on our CITE-seq data, and we observed clear agreement between the sorted bulk profiles and CITE-seq clusters (Figures 4D and 4E). Notably, our flow cytometry experiments utilized the exact antibody clones represented in the CITE-seq experiment, which can help to ensure that the two assays will return concordant results. We report each of these panels in Table S2 to facilitate similar experiments for additional clusters in our dataset. We note that although these panels can achieve high levels of enrichment, even optimally sorted groups may contain a minority of contaminating cells from other states. We show precision and recall metrics for each panel in Figure S4, demonstrating that it remains challenging to sort truly “homogeneous” populations of high-resolution subsets using a small number of markers.

### Multimodal heterogeneity within lymphoid populations

**Figure 5:**
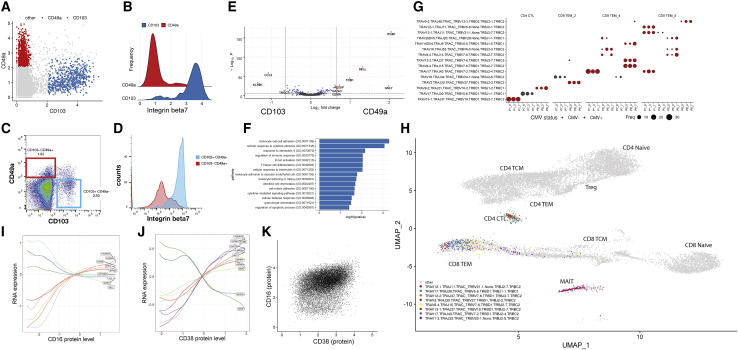
Characterizing heterogeneity within lymphoid populations (A) Mutually exclusive expression of the integrin proteins CD103 and CD49a within CD8^+^ T memory cells, as measured by CITE-seq. (B) Differential expression of integrin-7 between CD103^+^ CD49a^−^ and CD103^+^ CD49^+^ populations as measured by CITE-seq. (C and D) Flow cytometry validates the presence of these populations. Plots are the same as in (A) and (B) but generated via flow cytometry. (E and F) Differentially expressed genes, and enriched gene ontology terms, between CD103^+^ CD49a^−^ and CD103^−^ CD49^+^ populations. (G) Dot plot showing the representation of the fifteen most abundant T cell clonotypes in the dataset. For space, only the VDJ regions are shown on the y axis, but all cells in a clone share identical CDR3 sequences. Clones reside in a restricted set of cytotoxic and effector cell states and are shared across vaccination time points. Size of each dot represents the number of cells in the clonotype. Clones present in donors who were classified as CMV-positive are colored in red. (H) Cells within a clone exhibit similar molecular profiles. Grey dots represent T cells where TCR sequence was measured using the 10x 5′ assay. Cells from the eight most highly represented clonotypes are highlighted as colored dots. (I–K) Heterogeneity in NK cells is defined by two gradients correlating with CD16 and CD38 protein expression. (I) NK cells are ordered by their quantitative expression of CD16 protein expression. Rolling averages for the expression of genes that correlate positively or negatively with CD16 are shown as smoothed lines. (J) same as (I) but for CD38. (K) CD38 and CD16 protein expression define two separate gradients and are uncorrelated in NK cells. See also Figure S5 and Table S3.

**Figure S5:**
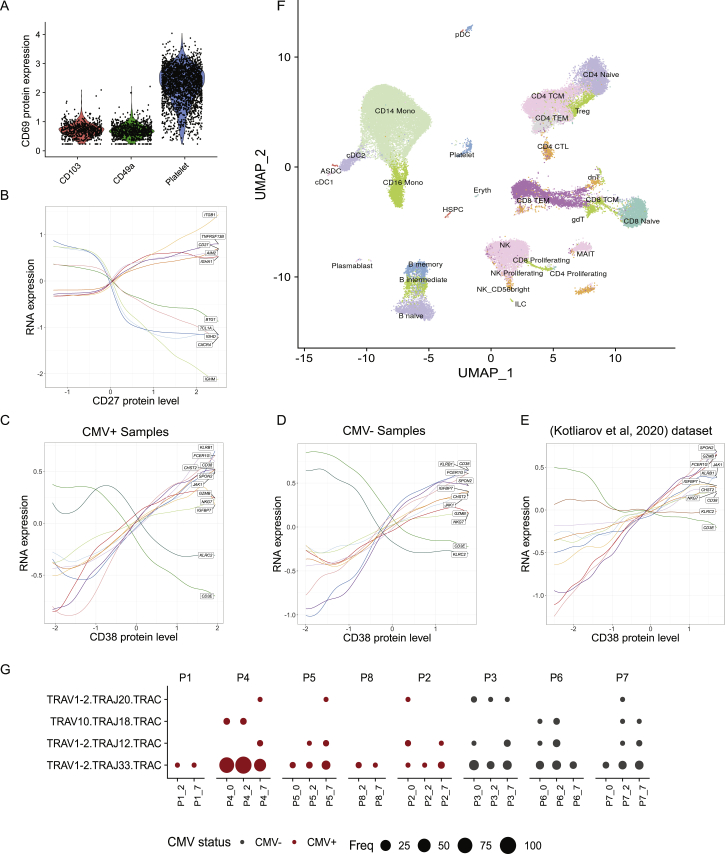
Additional heterogeneity within lymphoid populations, related to Figure 5 (A) Protein expression of canonical resident lymphocyte marker CD69 in CD8+ CD103+, CD8+ CD49a+ T cell populations. Neither population is positive. Platelets are included as a positive control, as CD69 is constitutively expressed on these cells. (B) Naive, intermediate and memory B cells are ordered by their quantitative level of CD27 protein expression. Rolling averages for the expression of genes that correlate positively or negatively with CD27 are shown as smoothed lines. (C-E) Same as Figure 5J, but after splitting the eight volunteers into five CMV+ (C) and three CMV- (D) samples (Table S3). We observe concordant trends in both subsets, as well as an independent CITE-seq dataset (Kotliarov et al., 2020). (F) UMAP visualization of CITE-seq dataset of 49,147 PBMC analyzed with the 10X 5′ Immune Profiling kit, which also measures immune repertoires. The dataset has been mapped onto the 3′-defined multimodal reference, allowing cells to be visualized in the same UMAP space as the reference, and cells are labeled based on transferred Level 2 annotations. (G) Dot plot showing the overrepresentation of TCRα sequences within cells annotated as MAIT. As expected, we detect the canonical MAIT TRAV1-2-TRAJ33 as the most abundant sequence along with reduced usage of TRAJ12 and TRAJ20. We also detect rare populations of invariant NKT cells (defined by the use of TRAV10.TRAJ18). As expected, and in contrast to the clonotypes reported in Figure 5G, these findings are consistent across volunteers, vaccination time points, and CMV status.

Our integrated WNN analysis reveals a rich diversity of T cell states that is not typically captured in scRNA-seq analyses, including CD4^+^ regulatory T cells, MAIT cells, multiple subpopulations of γδ and double-negative T cells, along with heterogeneous subpopulations of naive, memory, and effector states. Within CD8^+^ memory T cells, we identified distinct subpopulations defined by bimodal and mutually exclusive expression of the integrin proteins CD49a and CD103 (Figure 5A). Although we identified these cells in peripheral blood, expression of these proteins has traditionally been strongly associated with tissue-resident memory (TRM) cells, where integrins help mediate adhesion to epithelial cells or the extracellular matrix (Corgnac et al., 2018; Topham and Reilly, 2018). CD8^+^ CD103^+^ T cells expressed high surface protein levels of the heterodimeric co-binding partner integrin beta-7 (Figure 5B), while expression was absent in CD8^+^ CD49a^+^ groups. We validated the presence of the populations in independent healthy PBMC samples by performing flow cytometry for the same markers (Figures 5C and 5D). In addition, we identified modules of differentially expressed genes between these two groups (Figure 5E), which were enriched for T cell activation, differentiation, signaling response, and chemotaxis modules (Figure 5F). Both populations did not express the canonical resident marker CD69 (Szabo et al., 2019; Walsh et al., 2019) (Figure S5), suggesting that they are not TRMs that have temporarily detached and re-entered circulation. Instead, these subpopulations may represent cells that are preparing to become tissue-resident and have already begun to acquire distinguishing molecular characteristics.

In addition to characterizing heterogeneity in mRNA and protein expression, we leveraged our 5′ dataset to explore the relationship between molecular state and TCR sequence (STAR Methods). Overall clonal diversity was consistent across vaccination time points, consistent with an expected lack of a lymphoid response to vaccination within 7 days, and 97% of clones consisted only of a single cell. However, we also observed the presence of expanded clonal populations. As a positive control, we observed populations with highly restricted usage of TCRα sequences: both MAIT and invariant NKT cells exhibited closely related transcriptional profiles (Huang et al., 2019) and semi-invariant repertoires across multiple volunteers (Figure S5).

Excluding these populations, we identified 31 additional expanded clones consisting of at least 10 cells (Figure 5G). In each case, cells within a clonal population exhibited extremely similar molecular profiles (Figure 5H), representing subgroups of CD8^+^ T cells (primarily within our previously identified CD8_TEM_4 and CD 8_TEM_5 clusters), as well as cytotoxic CD4^+^ T cells (CD4 CTL). Each clone typically represented cells from a single volunteer, but could be independently found across multiple time points, including before vaccination (Figure 5G). Because our sample volunteers were generally middle-aged and otherwise healthy, we considered the possibility that overexpanded clones could be related to cytomegalovirus (CMV) infection (Kim et al., 2015). We assessed the CMV status of each volunteer by stimulating PBMCs with a CMV peptide pool and performing intracellular cytokine staining to determine responses in CD8^+^ T cells (Table S3; STAR Methods), identifying five positive and three negative volunteers. We found that the five positive samples accounted for 91% of cells within expanded clones.

We note that although WNN integration improves the ability to discover distinct cell subpopulations, it can also improve the characterization of cellular trajectories and continuous sources of heterogeneity. For example, within B cells, we identified a continuous trajectory connecting naive to memory cells defined by the canonical protein markers immunoglobulin D (IgD) and CD27, along with a module of correlated genes (Figure S5). Similarly, NK cells were subdivided into five clusters, representing variation across a continuous landscape. Our data show that the traditional division of NK cells into CD56-bright and CD56-dim categories represents a broader continuum defined by CD16 expression, alongside a module of genes that modulate cytotoxicity and correlate both positively and negatively with this marker (Figure 5I).

We also observed a second gradient defined by CD38 expression that, to our knowledge, has not been previously described. Notably, *KLRC2*, which encodes the NK activating receptor NKG2C was negatively associated with this continuum, while the signaling adaptor *FCER1G* was positively associated (Figure 5J). This expression pattern is consistent with the development of “adaptive” or “memory-like” NK cells observed in CMV seropositive individuals (Lee et al., 2015; Schlums et al., 2015). Notably, we observed consistent trends when restricting our analysis only to individuals with either positive or negative CMV T cell responses (Figure S5). We also observed consistent results (Figure S5) in an independent CITE-seq dataset of human PBMCs (Kotliarov et al., 2020). Our results indicate that this phenotype does not represent a strictly binary phenomenon and may not be specific to CMV response. Finally, we observed minimal correlation between CD38 and CD16 expression (Figure 5K), demonstrating that NK cells fall along a two-dimensional gradient defined by these markers.

Taken together, these results demonstrate that our dataset represents a powerful resource to enumerate cell states in the immune system, identify optimal reagents for cell-type-specific enrichment, and to understand the molecular heterogeneity in clonally related or antigen-specific cell groups. Because these results are consistent in both pre- and post-vaccination time points, they likely describe general characteristics of the healthy immune system.

### Characterizing the initial innate response to vaccination

**Figure 6:**
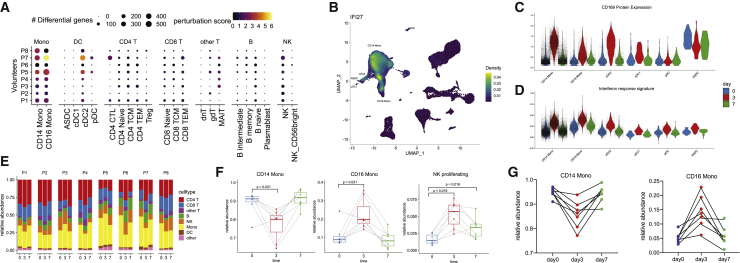
Identifying cell-type-specific responses to vaccination (A) For each of our level 2 annotated cell clusters, we calculated the number of differentially expressed genes between unvaccinated (day 0) and day 3 samples (size of each dot). As each per-gene test is highly sensitive to the number of cells, we also calculated a “perturbation score,” which reflects the strength of the molecular response based on the whole transcriptome (color of each dot). (B) Density plot, produced by the Nebulosa package, showing the expression of canonical interferon response gene IFI27. (C and D) Violin plot showing the protein upregulation of Siglec-1 (CD169) in single cells from day 3 samples (C), along with a signature of interferon response (D), in select cell types. In (A)–(D) we consistently observe robust responses only in CD14^+^ monocytes, CD16^+^ monocytes, and cDC2 DC. (E) Bar plot showing that the frequency of broad groups (level 1 annotations) is stable across the vaccination time course. (F) Within these broad categories, the relative abundance of classical monocytes, nonclassical monocytes, and proliferating NK cells across the vaccination time course. p values are computed using a paired Wilcoxon test. (G) Relative abundance of monocyte populations as measured by flow cytometry. See also Figure S6.

**Figure S6:**
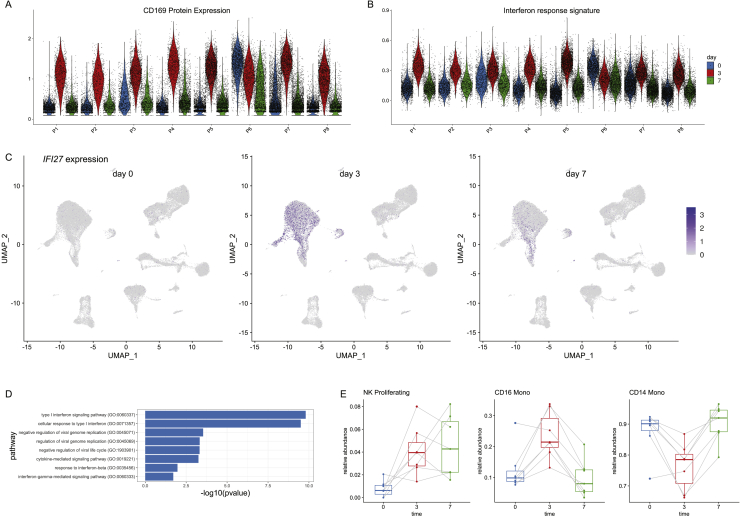
Cell-type-specific responses to vaccination, related to Figure 6 (A, B) Violin plot showing the upregulation of CD169 protein levels and a module of interferon response genes at day 3. Plot is similar to Figures 6C and 6D, but restricted to CD14 Monocytes, and shows the individual response of each volunteer. The response is consistent across all volunteers with one exception (P6), which exhibited signs of a highly activated immune system even prior to vaccination. (C) RNA expression of canonical interferon response gene IFI27 across the vaccination time course. The expression of IFI27 increases within particular myeloid populations at day 3, but dampens at day 7. (D) Pathway enrichment (enrichR) of the top DE genes between day 0 and day 3 myeloid cells exhibits a clear enrichment for components of the interferon response. (E) Same as in Figure 6F, but computed for cells profiled with the 10X 5′ kit.

We next explored our dataset to characterize the response to vaccination for each of our previously identified cell types. We were particularly interested to identify cell populations that contribute most strongly to the innate immune response, which is expected to be highly activated at our first vaccinated time point (day 3), and subsequently dampen in our second time point (day 7) as seen with another non-replicating viral vectored HIV vaccine (Zak et al., 2012). As expected, we observed robust responses in a subset of myeloid subpopulations, but only minimal responses in lymphoid groups (Figures 6A and 6B). Response patterns were also largely consistent across samples with the exception of one volunteer that exhibited a highly activated immune system in advance of vaccination and was removed from further analysis (Figure S6).

We observed the strongest changes in both CD14^+^ classical and CD16^+^ non-classical monocytes, as both cell types upregulated a shared module of 62 genes highly enriched for transcripts responsive to type I interferon (Figures 6A, 6B, and S6; visualization in Figure 6B from Alquicira-Hernandez and Powell [2020]). In addition, we identified Siglec-1 (CD169) as a protein response biomarker that was robustly induced only in day 3 samples (Figure 6C). When we examined dendritic cell populations, we observed a similarly robust response only within CD1C^+^ cDC2 cells. Contrastingly, closely related populations of CD141^+^ cDC1, as well as ASDC and pDC, exhibited minimal responses, and we did not detect any DE genes before and after vaccination for these groups (Figure 6A). This indicates that within DC subgroups, cDC2s may perform an important role in the downstream priming and activation of the adaptive immune system during this vaccine response.

We did not observe significant changes during the time course in overall abundance of broad immune classes (Figure 6E;); thus, we focused on identifying more subtle compositional changes. For example, although the overall proportion of monocytes was consistent across time points, there was a strong shift in the ratio between classical and non-classical populations between day 0 and day 3 (Figure 6F). We validated this result, as well as the observed return to baseline ratios at day 7, using flow cytometry on the same samples (Figure 6G). We did not observe changes within lymphoid cells with one exception: a small population of NK cells expressing proliferation and cell-cycle genes (NK_proliferation), consistently increased upon vaccination (Figure 6F). These findings were reproducible in independent analyses of the 3′ and 5′ scRNA-seq experiments and persisted in both day 3 and day 7 samples (Figures 6F and S6). This finding may reflect an early step in the development and maturation of NK cells, a key component of the NK cell-mediated innate immune response (Abel et al., 2018).

### Mapping query datasets to multimodal references

Single-cell transcriptomic profiling of the immune system has become routine, not only for healthy subjects, but also in multiple clinical contexts including for patients hospitalized with COVID-19. These datasets are typically processed using a workflow that consists of unsupervised clustering, which assumes minimal prior knowledge and is ideally suited for cell type discovery. However, having constructed a multimodal reference of the immune system, we sought to leverage this dataset to assist in the analysis and interpretation of additional single-cell experiments profiling human PBMCs (queries), even if only the transcriptome was profiled.

We first apply a procedure known as “supervised principal component analysis” (sPCA) (Barshan et al., 2011) to the transcriptome measurements in our reference dataset. Instead of seeking to identify a low-dimensional projection that maximizes total variance as in PCA, sPCA identifies a projection of the transcriptome dataset that maximally captures the structure defined in the WNN graph. Formally, given a gene expression matrix X and a WNN graph Y, sPCA identifies the transformation matrix U that maximizes the Hilbert-Schmidt Independence Criterion measure between a linear kernel of U^T^X and Y (STAR Methods). Informally, sPCA allows the weighted transcriptome and protein measurements to help “supervise” the analysis of gene expression data and identify the optimal transcriptomic vectors (gene modules) that define the cell states in our multimodal reference.

We compute this sPCA transformation on our reference (where both mRNA and protein were measured simultaneously), but can subsequently rapidly project this transformation onto any scRNA-seq query dataset. Combining this transformation with our previously described “anchor”-based framework (Stuart et al., 2019) allows us to place each scRNA-seq query cell on the previously defined reference UMAP visualization (STAR Methods) and annotate its identity based on reference clusters.

**Figure S7:**
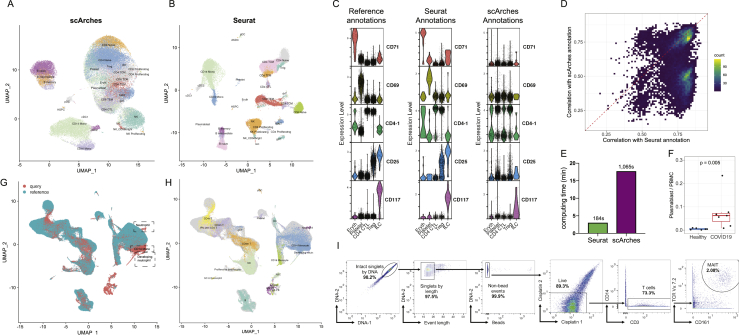
Reference-based mapping of query datasets, related to Figure 7 (A-E) Benchmarking of Seurat v4 reference-based mapping with scArches. Both methods utilize reference datasets to assist in the interpretation of query data. (A-B) UMAP visualizations of reference-based mapping of a human PBMC CITE-seq dataset from Kotliarov et al. (2020). Cells are label by the annotation that was transferred using each method. The protein data was withheld from mapping and can be used to assess accuracy. (C) For five cell types where we observed a high rate of discordant predictions between Seurat and scArches, we visualize the protein expression of key markers in the reference dataset (left), Seurat-transferred annotations (middle), and scArches-transferred annotations (right). In each case, the Seurat annotations provide the most concordant results. For example, cells annotated by Seurat as Treg express CD25 protein, while cells annotated by scArches as Treg do not. (D) For all 17,480 (32.9%) of query cells where Seurat and scArches returned different annotations based on the transcriptome, we calculated protein-based classification metrics to determine the support for each result (STAR Methods). In 73.8% of cases, we observe stronger support for the Seurat annotation. (E) Computing time for reference-mapping of Kotliarov et al. (2020) onto the multimodal reference. (F) The abundance of plamablasts increases during COVID-19 response. p value is computed using an unpaired Wilcoxon test. Annotations were derived from reference-based mapping, and confirm the result reported in Wilk et al. (2020). (G) ‘*de novo*’ UMAP (STAR Methods) visualization of the dataset from Wilk et al. (2020) after reference-mapping. Concordant cell types are identified between query and reference data with three exceptions, denoted with dashed rectangles. (H) Same as in (G), but cells are colored by their unsupervised label as described in Wilk et al. (2020). These results demonstrate that developing and differentiated neutrophils, which are not present in the reference, remain distinct after reference-based mapping. Additionally, a population of CD14+ Monocytes that has severe transcriptional responses to COVID-19 is also highlighted in this analysis. (I) Gating strategy used to identify MAIT cells in mass cytometry experiments.

**Figure 7:**
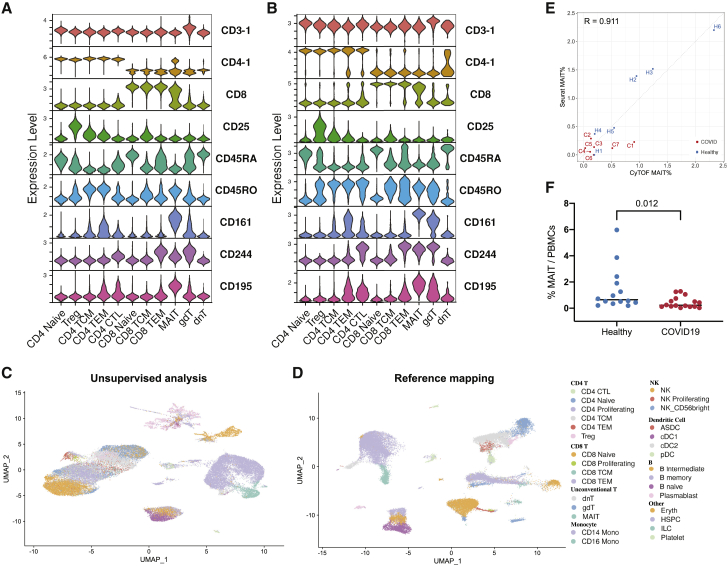
Supervised mapping of immune perturbations (A) Violin plots showing the expression patterns for nine proteins in our CITE-seq dataset. Cells are grouped by their WNN-defined T cell level 2 annotations. (B) Violin plots for the same proteins in an independent CITE-seq dataset of human PBMC (Kotliarov et al., 2020). Cells are grouped based on their predicted annotations from transcriptome-based reference mapping. The protein data were withheld from the mapping but displays the same patterns as in (A). (C) UMAP visualization of Wilk et al. (2020) scRNA-seq dataset, which includes 44,721 PBMC from patients hospitalized with COVID-19 and healthy controls. UMAP was computed using unsupervised analysis. (D) Same as in (C), but after the dataset has been mapped onto our multimodal reference. Cells are colored by their predicted level-2 annotations. (E) Quantification of MAIT cell abundance based on scRNA-seq reference mapping (y axis) and CyTOF (x axis) for the samples in Wilk et al. (2020). The Pearson correlation between these two methods is 0.911. (F) CyTOF quantification of MAIT cell abundance in PBMC samples from COVID-19 patients and healthy controls. p values are computed using an unpaired Wilcoxon test. See also Figure S7.

We found that this supervised mapping procedure dramatically improved our ability to analyze and interpret query scRNA-seq datasets compared to unsupervised analysis. We examined a recently generated dataset of human PBMCs prior to flu vaccination, which measured the transcriptomes of 53,099 cells alongside 82 surface proteins. We mapped this dataset onto our reference using only the transcriptome data and transferred our level 2 annotations, revealing the presence of multiple high-resolution lymphoid subsets (Figure S7). We verified the accuracy of our predictions using the query protein data, which was held out of the reference mapping procedure, yet revealed expression patterns based on our predicted annotations that were fully concordant with our reference dataset. For example, cells that were annotated as regulatory T cells expressed CD25 in the CITE-seq data, and we observed similar results for MAIT cells (CD161^+^), memory (CD45RA^−^ CD45RO^+^) and naive (CD45RA^+^ CD45RO^−^) T cells, and circulating ILC (CD117^+^ CD25^+^) (Figure S7). We benchmarked our method against scArches, a recently developed method for mapping scRNA-seq queries to reference datasets (Lotfollahi et al., 2020) and observed that our approach yielded substantial improvements in accuracy and performance (Figures 7A, 7B, and S7).

We next applied our mapping approach to a recent scRNA-seq study analyzing PBMC samples from seven patients hospitalized with COVID-19, alongside six healthy controls (Wilk et al., 2020). The original publication performed unsupervised clustering on the full dataset and identified six T cell clusters (three CD4^+^ T, two CD8^+^ T, and γδ T cells). In our supervised analysis, we transferred our level 2 annotations, successfully dividing T cells into the 12 groups (Figure 7C, D). Notably, populations of developing and differentiated neutrophils, which were identified by the original manuscript as being uniquely present in COVID-19 samples but were absent from our reference, could not be successfully mapped (Figure S7).

We leveraged our supervised annotations to test for differences in cell type abundance across disease conditions. Our findings recapitulated the original unsupervised analysis, for example, highlighting increases in plasmablast frequency during COVID-19 response (Figure S7). However, we also observed proportional shifts in cell states that were not detected in unsupervised clustering but were successfully annotated after reference mapping. In particular, we observed a depletion of MAIT cells in COVID-19 samples compared to healthy controls. To validate our findings, we performed CyTOF on both the original samples and a validation cohort of 16 additional samples. We observed strong quantitative agreement (R = 0.911) in the fraction of MAIT cells predicted by scRNA-seq and measured by CyTOF in the original cohort (Figure 7E). Moreover, CyTOF analysis of the larger sample set identified a depletion of MAIT cells in COVID-19 samples (Figures 7F and S7). This change in abundance may reflect these cells exiting circulation to play protective roles in barrier tissues during the antiviral immune response (Grimaldi et al., 2014; Hinks and Zhang, 2020; Provine and Klenerman, 2020).

## Discussion

In order to leverage multiple data types to define cellular identity, we developed WNN analysis, a computational method that learns the information content of each modality and generates an integrated representation of multimodal data. By calculating cell-specific modality weights, WNN analysis solves an important technical challenge for the analysis of multimodal datasets and allows for flexible application across a range of modalities and data types. We demonstrate throughout this manuscript that performing downstream analyses on a weighted combination of data types improves our ability to characterize cellular diversity.

We apply our approach to analyze a dataset of human PBMCs featuring paired transcriptomes and measurements of 228 surface proteins, representing a multimodal atlas of the immune system. We leverage this resource to characterize extensive lymphoid heterogeneity that has not been previously observed by scRNA-seq alone, including the heterogeneous expression of integrin proteins on circulating memory T cells, a gradient of adaptive-like responses in NK cells, and tightly clustered clonal populations within effector and cytotoxic groups. Our data also enable us to explore the response of the innate immune system to vaccination, highlighting specific response biomarkers, as well as the heterogeneous responses of conventional DCs. Importantly, we demonstrate that CITE-seq data can be easily mined to identify the best immunophenotypic marker panels for any subpopulation of interest. These marker panels can be used for flow cytometry with the same antibody clones in our CITE-seq panel, facilitating rapid enrichment and downstream analysis of these groups, and broadening the value of our resource.

In addition to constructing a multimodal reference, we demonstrate the ability to map scRNA-seq data onto this dataset. We accomplish this via a supervised version of principal component analysis to identify the best transcriptomic modules which delineate our WNN-defined cell types. Supervised mapping represents an attractive alternative to unsupervised analysis, and we show how this workflow can improve cell type identification and robustly integrate samples from multiple donors and disease states. To assist the community in utilizing our resource, we have created a web application, freely available at https://azimuth.hubmapconsortium.org/, which enables users to rapidly map their own datasets online, automating the process of visualization and annotation. Using this approach, a dataset of 50,000 cells can be fully processed and mapped in less than 5 min. As the profiling of human PBMCs under a variety of disease states becomes increasingly routine, the ability to perform automated mapping of these datasets will facilitate the characterization of complex immune responses, and the discovery of pathogenic populations. We note that our supervised mapping is not limited to scRNA-seq and can be extended to other modalities. For example, future extensions of this work could modify our supervised PCA procedure to identify optimal transformations of multiplexed protein measurements. This would enable the mapping of mass cytometry profiles to our multimodal reference, even in the absence of transcriptomic data.

Last, we note that the modality weights learned in our procedure serve not only as a proxy for the technical quality of a measurement type, but may also reflect the biological importance of each modality in determining cellular identity. For example, our analyses of human bone marrow demonstrated that progenitor cells and differentiated cells exhibited divergent modality weights. As future technologies enable the simultaneous measurement of modalities spanning the central dogma including chromatin state, DNA methylation, transcription, lineage, spatial location, and protein levels—WNN analysis may help to reveal how subpopulations of cells differentially utilize these modalities to regulate their current state and future potential. Our current implementation of WNN analysis extends to handle three or more simultaneously measured modalities, as these technologies mature. Integrative multimodal analysis therefore provides a path forward to move beyond the partial and transcriptome-focused view of a cell and toward a unified definition of cellular behavior, identity, and function.

### Limitations of the study

We note the following limitations with our study. First, WNN requires a dimensional reduction to describe the neighborhood structure between cells. This requirement is not compatible with categorical or low-dimensional data. Second, WNN assumes that modalities do not define conflicting sets of cell states. Although we have not observed this when using molecular data such as chromatin state, gene expression, and surface protein abundance, this assumption may be problematic when integrating morphological, functional, and molecular data. In addition, our circulating immune atlas was constructed from PBMCs and therefore contains few cells with no nuclei (erythrocytes) or multi-lobed nuclei (granulocytes).

## STAR★Methods

### Key resources table

**Table:** 

REAGENT or RESOURCE	SOURCE	IDENTIFIER
**Antibodies**		

B7-H4	BioLegend TotalSeq-A	Cat# 358114
C5L2	BioLegend TotalSeq-A	Cat# 342407
Cadherin	BioLegend TotalSeq-A	Cat# 368715
CCR10	Custom made	clone 6588-5
CD102	BioLegend TotalSeq-A	Cat# 328509
CD103	BioLegend TotalSeq-A	Cat# 350231
CD105	BioLegend TotalSeq-A	Cat# 323221
CD106	BioLegend TotalSeq-A	Cat# 305813
CD107a	BioLegend TotalSeq-A	Cat# 328647
CD109	BioLegend TotalSeq-A	Cat# 323307
CD110	Custom made	clone S16017E
CD112	BioLegend TotalSeq-A	Cat# 337417
CD115	BioLegend TotalSeq-A	Cat# 347325
CD117	BioLegend TotalSeq-A	Cat# 313241
CD119	BioLegend TotalSeq-A	Cat# 308607
CD11a/CD18	BioLegend TotalSeq-A	Cat# 363425
CD11b_1	BioLegend TotalSeq-A	Cat# 101265
CD11b_2	BioLegend TotalSeq-A	Cat# 301353
CD11c	BioLegend TotalSeq-A	Cat# 371519
CD122	Custom made	clone TU27
CD123	BioLegend TotalSeq-A	Cat# 306037
CD124	Custom made	clone G077F6
CD126	BioLegend TotalSeq-A	Cat# 352813
CD127	BioLegend TotalSeq-A	Cat# 351352
CD13	BioLegend TotalSeq-A	Cat# 301729
CD133_1	BioLegend TotalSeq-A	Cat# 372815
CD133_2	BioLegend TotalSeq-A	Cat# 394005
CD134	BioLegend TotalSeq-A	Cat# 350033
CD135	BioLegend TotalSeq-A	Cat# 313317
CD137	BioLegend TotalSeq-A	Cat# 309835
CD138_1	BioLegend TotalSeq-A	Cat# 356533
CD138_2	BioLegend TotalSeq-A	Cat# 352325
CD14	BioLegend TotalSeq-A	Cat# 301855
CD140a	BioLegend TotalSeq-A	Cat# 323509
CD140b	BioLegend TotalSeq-A	Cat# 323609
CD141	BioLegend TotalSeq-A	Cat# 344121
CD142	BioLegend TotalSeq-A	Cat# 365207
CD144	BioLegend TotalSeq-A	Cat# 348517
CD146	BioLegend TotalSeq-A	Cat# 361017
CD15	BioLegend TotalSeq-A	Cat# 323046
CD152	BioLegend TotalSeq-A	Cat# 369619
CD154	BioLegend TotalSeq-A	Cat# 310843
CD155	BioLegend TotalSeq-A	Cat# 337623
CD158	BioLegend TotalSeq-A	Cat# 339515
CD158b	BioLegend TotalSeq-A	Cat# 312615
CD158e1	BioLegend TotalSeq-A	Cat# 312723
CD158f	BioLegend TotalSeq-A	Cat# 341307
CD16	BioLegend TotalSeq-A	Cat# 302061
CD161	BioLegend TotalSeq-A	Cat# 339945
CD163	BioLegend TotalSeq-A	Cat# 333635
CD164	BioLegend TotalSeq-A	Cat# 324809
CD169	BioLegend TotalSeq-A	Cat# 346011
CD171	BioLegend TotalSeq-A	Cat# 371609
CD172a	BioLegend TotalSeq-A	Cat# 372109
CD177	BioLegend TotalSeq-A	Cat# 315811
CD178	BioLegend TotalSeq-A	Cat# 306413
CD18	BioLegend TotalSeq-A	Cat# 302121
CD184	BioLegend TotalSeq-A	Cat# 306531
CD185	BioLegend TotalSeq-A	Cat# 356937
CD186	BioLegend TotalSeq-A	Cat# 356021
CD19	BioLegend TotalSeq-A	Cat# 302259
CD192	BioLegend TotalSeq-A	Cat# 357229
CD193	BioLegend TotalSeq-A	Cat# 310729
CD194	BioLegend TotalSeq-A	Cat# 359423
CD195	BioLegend TotalSeq-A	Cat# 359135
CD196	BioLegend TotalSeq-A	Cat# 353437
CD199	BioLegend TotalSeq-A	Cat# 358919
CD1a	BioLegend TotalSeq-A	Cat# 300133
CD1c	BioLegend TotalSeq-A	Cat# 331539
CD1d	BioLegend TotalSeq-A	Cat# 350317
CD2	BioLegend TotalSeq-A	Cat# 309229
CD20	BioLegend TotalSeq-A	Cat# 302359
CD200	Custom made	clone OX-104
CD201	BioLegend TotalSeq-A	Cat# 351907
CD202b	BioLegend TotalSeq-A	Cat# 334213
CD203c	BioLegend TotalSeq-A	Cat# 324627
CD204	BioLegend TotalSeq-A	Cat# 371909
CD205	BioLegend TotalSeq-A	Cat# 342211
CD206	BioLegend TotalSeq-A	Cat# 321143
CD207	BioLegend TotalSeq-A	Cat# 352207
CD209	BioLegend TotalSeq-A	Cat# 330119
CD21	BioLegend TotalSeq-A	Cat# 354915
CD22	BioLegend TotalSeq-A	Cat# 363514
CD223	BioLegend TotalSeq-A	Cat# 369333
CD226	BioLegend TotalSeq-A	Cat# 337111
CD235a	BioLegend TotalSeq-A	Cat# 349117
CD235ab	BioLegend TotalSeq-A	Cat# 306623
CD24	BioLegend TotalSeq-A	Cat# 311137
CD243	BioLegend TotalSeq-A	Cat# 919407
CD244	BioLegend TotalSeq-A	Cat# 329527
CD25	BioLegend TotalSeq-A	Cat# 302643
CD252	Custom made	clone 11C3.1
CD253	BioLegend TotalSeq-A	Cat# 308211
CD26_1	BioLegend TotalSeq-A	Cat# 302720
CD26_2	Custom made	clone BA5b
CD267	BioLegend TotalSeq-A	Cat# 311913
CD268	BioLegend TotalSeq-A	Cat# 316925
CD269	BioLegend TotalSeq-A	Cat# 357521
CD27	BioLegend TotalSeq-A	Cat# 302847
CD270	BioLegend TotalSeq-A	Cat# 318813
CD271	BioLegend TotalSeq-A	Cat# 345123
CD272	BioLegend TotalSeq-A	Cat# 344525
CD273	BioLegend TotalSeq-A	Cat# 329619
CD274	BioLegend TotalSeq-A	Cat# 329743
CD275_1	BioLegend TotalSeq-A	Cat# 309413
CD275_2	BioLegend TotalSeq-A	Cat# 329809
CD278	BioLegend TotalSeq-A	Cat# 313555
CD279	BioLegend TotalSeq-A	Cat# 329955
CD28	BioLegend TotalSeq-A	Cat# 302955
CD284	BioLegend TotalSeq-A	Cat# 312817
CD29	BioLegend TotalSeq-A	Cat# 303027
CD294	BioLegend TotalSeq-A	Cat# 350127
CD3_1	BioLegend TotalSeq-A	Cat# 300475
CD3_2	BioLegend TotalSeq-A	Cat# 344847
CD30	BioLegend TotalSeq-A	Cat# 333913
CD301	BioLegend TotalSeq-A	Cat# 354707
CD303	BioLegend TotalSeq-A	Cat# 354239
CD304	BioLegend TotalSeq-A	Cat# 354525
CD305	BioLegend TotalSeq-A	Cat# 342805
CD307c/FcRL3	BioLegend TotalSeq-A	Cat# 374411
CD307d	BioLegend TotalSeq-A	Cat# 340209
CD307e	BioLegend TotalSeq-A	Cat# 340307
CD309	BioLegend TotalSeq-A	Cat# 359919
CD31	BioLegend TotalSeq-A	Cat# 303137
CD314	BioLegend TotalSeq-A	Cat# 320835
CD319	BioLegend TotalSeq-A	Cat# 331821
CD324	BioLegend TotalSeq-A	Cat# 324125
CD325	BioLegend TotalSeq-A	Cat# 350817
CD335	BioLegend TotalSeq-A	Cat# 331943
CD337	BioLegend TotalSeq-A	Cat# 325221
CD338	BioLegend TotalSeq-A	Cat# 332021
CD34	BioLegend TotalSeq-A	Cat# 343537
CD340	BioLegend TotalSeq-A	Cat# 324423
CD35	BioLegend TotalSeq-A	Cat# 333407
CD354	Custom made	clone TREM-26
CD357	BioLegend TotalSeq-A	Cat# 371225
CD36	BioLegend TotalSeq-A	Cat# 336225
CD366	BioLegend TotalSeq-A	Cat# 345047
CD370	BioLegend TotalSeq-A	Cat# 353807
CD38_1	BioLegend TotalSeq-A	Cat# 303541
CD38_2	BioLegend TotalSeq-A	Cat# 356635
CD39	BioLegend TotalSeq-A	Cat# 328233
CD4_1	BioLegend TotalSeq-A	Cat# 344649
CD4_2	BioLegend TotalSeq-A	Cat# 300563
CD40	BioLegend TotalSeq-A	Cat# 334346
CD41	BioLegend TotalSeq-A	Cat# 303737
CD42b	BioLegend TotalSeq-A	Cat# 303937
CD43	BioLegend TotalSeq-A	Cat# 343209
CD44_1	BioLegend TotalSeq-A	Cat# 103045
CD44_2	BioLegend TotalSeq-A	Cat# 338825
CD45_1	BioLegend TotalSeq-A	Cat# 368543
CD45_2	BioLegend TotalSeq-A	Cat# 304064
CD45RA	BioLegend TotalSeq-A	Cat# 304157
CD45RB	BioLegend TotalSeq-A	Cat# 310209
CD45RO	BioLegend TotalSeq-A	Cat# 304255
CD46	BioLegend TotalSeq-A	Cat# 352415
CD47	BioLegend TotalSeq-A	Cat# 323129
CD48	BioLegend TotalSeq-A	Cat# 336709
CD49a	BioLegend TotalSeq-A	Cat# 328315
CD49b	BioLegend TotalSeq-A	Cat# 359311
CD49d	BioLegend TotalSeq-A	Cat# 304337
CD52	BioLegend TotalSeq-A	Cat# 316017
CD54	BioLegend TotalSeq-A	Cat# 353123
CD55	BioLegend TotalSeq-A	Cat# 311317
CD56_1	BioLegend TotalSeq-A	Cat# 362557
CD56_2	BioLegend TotalSeq-A	Cat# 392421
CD57	BioLegend TotalSeq-A	Cat# 393319
CD59	BioLegend TotalSeq-A	Cat# 304709
CD61	BioLegend TotalSeq-A	Cat# 336423
CD62E	BioLegend TotalSeq-A	Cat# 336017
CD62P	BioLegend TotalSeq-A	Cat# 304933
CD63	BioLegend TotalSeq-A	Cat# 353035
CD64	BioLegend TotalSeq-A	Cat# 305037
CD66a/c/e	BioLegend TotalSeq-A	Cat# 342319
CD66b	BioLegend TotalSeq-A	Cat# 392905
CD68	Custom made	clone Y1/82A
CD69	BioLegend TotalSeq-A	Cat# 310947
CD70	BioLegend TotalSeq-A	Cat# 355117
CD71	BioLegend TotalSeq-A	Cat# 334123
CD72	BioLegend TotalSeq-A	Cat# 316205
CD73	BioLegend TotalSeq-A	Cat# 344029
CD79a	Custom made	clone HM47
CD79b	BioLegend TotalSeq-A	Cat# 341415
CD8	BioLegend TotalSeq-A	Cat# 344751
CD80	BioLegend TotalSeq-A	Cat# 305239
CD81	BioLegend TotalSeq-A	Cat# 349521
CD83	BioLegend TotalSeq-A	Cat# 305339
CD85 g	BioLegend TotalSeq-A	Cat# 326411
CD86	BioLegend TotalSeq-A	Cat# 305443
CD8a	BioLegend TotalSeq-A	Cat# 301067
CD9	BioLegend TotalSeq-A	Cat# 312119
CD90	BioLegend TotalSeq-A	Cat# 328135
CD93	BioLegend TotalSeq-A	Cat# 336121
CD95	BioLegend TotalSeq-A	Cat# 305649
CD96	BioLegend TotalSeq-A	Cat# 338419
CD98	BioLegend TotalSeq-A	Cat# 315605
CD99	BioLegend TotalSeq-A	Cat# 371317
CLEC12A	BioLegend TotalSeq-A	Cat# 353613
CLEC2	BioLegend TotalSeq-A	Cat# 372009
CX3CR1	BioLegend TotalSeq-A	Cat# 355709
Folate	BioLegend TotalSeq-A	Cat# 391707
Galectin-9	Custom made	clone 9M1-3
GP130	Custom made	clone 2E1B02
HLA-DR	BioLegend TotalSeq-A	Cat# 307659
IgD	BioLegend TotalSeq-A	Cat# 348243
IgM	BioLegend TotalSeq-A	Cat# 314541
Integrin_7	BioLegend TotalSeq-A	Cat# 321227
LOX-1	BioLegend TotalSeq-A	Cat# 358611
MERTK	BioLegend TotalSeq-A	Cat# 367617
Notch_1	BioLegend TotalSeq-A	Cat# 352109
Notch_2	BioLegend TotalSeq-A	Cat# 345411
Podoplanin	BioLegend TotalSeq-A	Cat# 337019
Rag_IgG2c	BioLegend TotalSeq-A	Cat# 400739
Rat_IgG1_1	BioLegend TotalSeq-A	Cat# 400459
Rat_IgG1_2	BioLegend TotalSeq-A	Cat# 401919
Rat_IgG2b	BioLegend TotalSeq-A	Cat# 400673
Siglec-8	Custom made	clone 7C9
TCR_1	BioLegend TotalSeq-A	Cat# 331229
TCR_2	BioLegend TotalSeq-A	Cat# 306737
TCR_V_2	BioLegend TotalSeq-A	Cat# 331433
TCR_V_24_J_18	BioLegend TotalSeq-A	Cat# 342923
TCR_V_7.2	BioLegend TotalSeq-A	Cat# 351733
TCR_V_9	BioLegend TotalSeq-A	Cat# 331311
TIGIT	BioLegend TotalSeq-A	Cat# 372725
TIM-4	BioLegend TotalSeq-A	Cat# 354009
TSLPR	BioLegend TotalSeq-A	Cat# 322907
VEGFR-3	Custom made	clone 9D9F9
XCR1	BioLegend TotalSeq-A	Cat# 372613
CD3	Custom made	clone UCHT1
CD4	Custom made	clone RPA-T4
CD8	Custom made	clone RPA-T8
TCRb	Custom made	clone IP26
TCRg	Custom made	clone B1
CD44	Custom made	clone BJ18
CD62L	Custom made	clone DREG-56
Ox40 (CD134)	Custom made	clone Ber-ACT35
PD1 (CD279)	Custom made	clone EH12.2H7
PD-L1	Custom made	clone 29E.2A3
EpCAM (CD326)	Custom made	clone 9C4
CD66b	Custom made	clone 6/40C
MHCII (HLA-DR)	Custom made	clone L243
CD45	Custom made	clone H130
CD19	Custom made	clone H1B19
B220 (CD45R)	Custom made	clone RA3-6B2
CD11c	Custom made	clone 3.9
CD14	Custom made	clone M5E2
CD34	Custom made	clone 581
CD56	Custom made	clone 5.1H11
CD16	Custom made	clone B73.1
CD2	Custom made	clone TS1/8
CD5	Custom made	clone UCHT2
CD45RA	Custom made	clone HI100
CD45RO	Custom made	clone UCHL1
CCR7	Custom made	clone GO43H7
CD11b	Custom made	clone ICRF44
CD1a	Custom made	clone HI149
CD27	Custom made	clone M-T271
CD69	Custom made	clone FN50
PECAM (CD31)	Custom made	clone WM59
CD138	Custom made	clone DL-101
CD24	Custom made	clone ML5
Siglec-8	Custom made	clone 7C9
LAMP1	Custom made	clone H4A3
C-kit (CD117)	Custom made	clone 104D2
IL7Ralpha (CD127)	Custom made	clone A019D5
CTLA4	Custom made	clone BNI3
HLA-A,B,C	Custom made	clone W6/32
CD77	Custom made	clone 5B5
CD366 (tim3)	Custom made	clone F38-2E2
CLA	Custom made	clone HECA-452
CD28	Custom made	clone CD28.2
CD7	Custom made	clone CD7-6B7
CD26 (Adenosine)	Custom made	clone BA5b
PD-1 (CD279)	Custom made	clone NAT105
PD-L1 (CD274)	Custom made	clone MIH1
CD161	Custom made	clone CD161
CD123	Custom made	clone CD123
CD25	Custom made	clone CD25
IgG1	Custom made	clone MOPC21
IgG2a	Custom made	clone MOPC273
CD45RA	Custom made	clone HI100
CD45RO	Custom made	clone UCHL1

**Biological samples**		

Human PBMC	Cape Town HVTN Immunology Lab, South Africa	https://www.chil.org.za/
Human PBMC	AllCells	Lot 3032552

**Critical commercial assays**		

3′ scRNA-seq kit	10x Genomics	v3 GEM kit
5′ VDJ-seq kit	10x Genomics	v1 V(D)J kit

**Deposited data**		

Human PBMC this paper – CITE-seq, ECITE-seq	This paper	GEO: GSE164378 dbGAP: phs002315.v1.p1
Human cord blood mononuclear cells – CITE-seq	Stoeckius et al., 2017	GEO: GSE100866
Human bone marrow mononuclear cells – CITE-seq	Stuart et al., 2019	GEO: GSE128639
Human PBMC – ASAP-seq	Mimitou et al., 2020	GEO: GSE156473
Human PBMC – 10x multiome ATAC+Gene expression	Single Cell Multiome ATAC + Gene Exp. Datasets	https://support.10xgenomics.com/single-cell-multiome-atac-gex/datasets
Mouse skin cells – SHARE-seq	Ma et al., 2020	GEO: GSE140203
Human PBMC–CITE-seq	Kotliarov et al., 2020	https://nih.figshare.com/collections/Data_and_software_code_repository_for_Broad_immune_activation_underlies_shared_set_point_signatures_for_vaccine_responsiveness_in_healthy_individuals_and_disease_activity_in_patients_with_lupus_Kotliarov_Y_Sparks_R_et_al_Nat_Med_DOI_https_d/4753772
Human PBMC – scRNA-seq	Wilk et al., 2020	https://www.covid19cellatlas.org/index.patient.html

**Software and algorithms**		

Seurat v4	This paper	https://github.com/satijalab/seurat
Azimuth	This paper	https://azimuth.hubmapconsortium.org/
Seurat v3.2.0	Stuart et al., 2019	https://github.com/satijalab/seurat/releases/tag/v3.2.0
SCTransform v0.3.2	Hafemeister and Satija, 2019	https://github.com/ChristophH/sctransform
Cell Ranger v3.1.0	10x Genomics	https://support.10xgenomics.com/single-cell-gene-expression/software/pipelines/latest/installation
Cell Ranger vdj v3.0.2	10x Genomics	https://support.10xgenomics.com/single-cell-gene-expression/software/pipelines/latest/installation
Salmon Alevin v1.3.0	Srivastava et al., 2019	https://salmon.readthedocs.io/en/latest/alevin.html
totalVI v0.6.7	Gayoso et al., 2019	https://github.com/YosefLab/scvi-tools
MOFA+ v1.1	Argelaguet et al., 2020	https://biofam.github.io/MOFA2
scArches v0.1.2	Lotfollahi et al., 2020	https://github.com/theislab/scarches
uwot v 0.1.10	McInnes et al., 2018	https://github.com/jlmelville/uwot
Presto v1.0.0	Korsunsky et al., 2019	https://github.com/immunogenomics/presto
Signac v1.0.0	Stuart et al., 2020	https://satijalab.org/signac/index.html
R	R Core	https://www.r-project.org/
Python	Python Software Foundation	https://www.python.org/

### Resource availability

#### Lead contact

Further information and requests for resources and reagents should be directed to and will be fulfilled by the Lead Contact, Rahul Satija (rsatija@nygenome.org)

#### Materials availability

No unique reagents were generated for this study.

#### Data and code availability

CITE-seq data generated for this manuscript is available to download and explore at https://atlas.fredhutch.org/nygc/multimodal-pbmc/. All CITE-seq and ECITE-seq raw data are available in GEO database under the accession number GEO: GSE164378. All raw sequencing data are deposited in the dbGaP under the accession number dbGaP: phs002315.v1.p1.

Seurat v4 is released under the open source GPLv3 license, and all code is available at https://www.github.com/satijalab/seurat. To facilitate the mapping of new query datasets to the multimodal PBMC reference described in this manuscript, we have released an automated web app, Azimuth: https://azimuth.hubmapconsortium.org/.

### Experimental model and subject details

#### HIV vaccine trial specimens

HVTN 087 (NCT01578889) was a phase 1a HIV vaccine trial that tested intramuscular electroporation of a DNA vaccine with or without IL-12 adjuvant delivered as a plasmid at months 0, 1 and 3 followed by boosting with VSV-vectored HIV *gag* vaccine at month 6 (Elizaga et al., 2018; Li et al., 2017). Eight participants in this trial were selected for single cell analysis from Group 1 (no IL-12) and Group 3 (1000 mcg IL-12) based on sample availability. Participant demographics and group assignments are listed in Table S1. Blood was collected immediately before the first VSV-Gag administration and at sequential time points afterward. PBMC were isolated and cryopreserved as previously described (Bull et al., 2007).

### Method details

#### Antibody titration, staining, and cleanup

For CITE-seq / TotalSeq-A / 3P scRNA-seq experiments, we pooled together 228 TotalSeq-A antibodies from BioLegend (Table S2). In preliminary experiments designed to test the balance of markers in the panel, reads corresponding to 12 antibodies each took up more than 2% of the total sequencing space, and combined together, accounted for half of the total antibody reads. The signal for each of these markers was reduced by addition of a proportional amount of unlabeled antibodies. We recommend the addition of unlabeled antibodies as an effective strategy to modify existing panels to be more robust to sequencing saturation from highly expressed protein markers. CITE-seq antibodies and unlabeled blocking antibodies were then combined in PBS and concentrated with a 50 kDa Amicon filter as per manufacturer’s instructions. Post elution, BSA was added to a final concentration of 2%.

For ECCITE-seq / TotalSeq-C / 5P scRNA-seq experiments, we used a combination of antibody:oligo conjugates designed for ECCITE-seq (Mimitou et al., 2019), conjugated as described (Stoeckius et al., 2018), and commercially available TotalSeq-C reagents. 52 ECCITE-seq antibodies (Mimitou et al., 2019) were pooled together at a concentration of 1 μg each per test, and combined with TotalSeq-C reagents for CD45RA and CD45RO at 0.25 μg each per test. Antibodies were pooled together and concentrated in a 50 kDa Amicon filter as per manufacturer’s instructions in PBS. Post elution, BSA was added to a final concentration of 2%.

#### CITE-seq staining and sample preparation

To minimize batch effects, for each experiment we processed frozen PBMCs from four different patients at 3 different time points (day 0, day 3, and day 7). After thawing, cells were incubated with FcX block (BioLegend) for 10 min. Cells were then divided into separate aliquots and processed independently for the 3P and 5P protocols.

For the 3P CITE-seq staining protocol, samples were stained simultaneously with the antibody/block pool and a unique hashtag for 30 min. Cells were then washed 3 times in staining buffer (2% BSA, 0.01% Tween in PBS) and filtered using a 40 μm Flowmi filter in PBS and pooled in equal proportions. Cells were loaded into 8 lanes of a 10x Genomics Chip B, at 45,000 cells per lane using the 10x Genomics 3′ v3 GEM kit.

For the 5P ECCITE-seq staining protocol, each sample of cells was first stained with a unique hashtag for 30 min. Cells were then washed 3 times in staining buffer, pooled together, and stained with the antibody panel for 30 min. The pool of cells was then washed 3 times in staining buffer and filtered using a 40μm Flowmi filter in PBS. Cells were loaded into 2 lanes of a 10x Genomics Chip A, at 45000 cells per lane, using the 10x Genomics V(D)J kit (v1).

For both 3P and 5P experiments, first strand cDNA was generated by incubating the emulsions according to the respective 10x Genomics protocol. Emulsions were then broken and nucleic acids recovered Subsequent library preparation steps are detailed in the section below.

#### Library prep

##### CITE-seq / 3P scRNA-seq

The 10x 3P v3 protocol was followed according to manufacturer’s instructions for cDNA amplification, with the following modifications:

• During cDNA amplification, 0.2 μM of ADT additive primer (**5′CCTTGGCACCCGAGAATTCC**) and 0.2 μM of HTO additive primer (5′GTGACTGGAGTTCAGACGTGTGCTC) were added to the reaction.
• During cDNA cleanup, the supernatant from the 0.6x SPRI cleanup was saved and purified with two rounds of 2x SPRI. The eluate was split and used as template for production of ADT and Hashtag libraries:
• Hashtag libraries were generated by PCR using Kapa Hifi Master Mix, 10 μM 10x Genomics SI-PCR primer (5′AATGATACGGCGACCACCGAGATCTACACTCTTTCCCTACACGACGCTC), and 10 μM Illumina TruSeq DNA D7xx primer (5′CAAGCAGAAGACGGCATACGAGATxxxxxxxxGTGACTGGAGTTCAGACGTGTGC). Following amplification, Hashtag libraries were and cleaned up with 1.6x SPRI.

Antibody tag libraries were generated by PCR using Kapa Hifi Master Mix, 10 μM 10x Genomics SI-PCR primer, and 10 μM TruSeq Small RNA RPIx primer (5′CAAGCAGAAGACGGCATACGAGxxxxxxxxGTGACTGGAGTTCCTTGGCACCCGAGAATTCCA) Following amplification, Antibody tag libraries were and cleaned up with 1.6x SPRI.

##### ECCITE-seq / 5P scRNA-seq / immune receptor:

The 10x Immune Profiling v1 protocol was followed according to manufacturer’s instructions for cDNA amplification, with the following modifications:

• During cDNA amplification, 0.2 μM each of ADT (**5′CCTTGGCACCCGAGAATT^∗^C^∗^C)**, HTO (**5′GTGACTGGAGTTCAGACGTGTGC^∗^T^∗^C)**, and TotalSeq-C additives (5′ CTCGTGGGCTCGGAGATGTGTATAAGAGACAG) were added to the reaction.
• Post cDNA cleanup, a 0.6x SPRI cleanup was performed, where larger cDNA fragments were kept on the beads, and the smaller tag libraries were retained in the supernatant. From the material retained on the beads, a portion of the eluted material was used to generate TCR α/β libraries (as written in the 10x protocol), BCR libraries (as written in the 10x protocol) and TCR γ/δ libraries (as written in the 10x protocol for TCR α/β, with these modifications):
• 5 μL of cDNA was taken into the initial reaction
• For the first PCR, instead of the TCR1 primer mix provided by 10x genomics, we substituted our own mix consisting of primers 5′AGCTTGACAGCATTGTACTTCC and 5′TGTGTCGTTAGTCTTCATGGTGTTCC
• For the second PCR, instead of the TCR2 primer mix provided by 10x Genomics, we substituted our own of primers consisting of 5′TCCTTCACCAGACAAGCGAC and 5′GATCCCAGAATCGTGTTGCTC
• The 0.6X SPRI supernatant remaining following cDNA cleanup was subjected to 2 rounds of 2x SPRI. The eluate was split into three reactions for tag library production:
• Hashtag libraries were created by performing a PCR reaction consisting of Kapa Hifi Master Mix, 10 μM 10x Genomics SI-PCR primer, and 10 μM Illumina TruSeq DNA D7xx primer.
• Antibody libraries (for homemade conjugates) were created by performing a PCR reaction with Kapa Hifi Master Mix, 10 μM 10x Genomics SI-PCR primer, and 10 μM TruSeq Small RNA RPIx primer.
• TotlaSeq-C antibody libraries were created by performing a PCR reaction with 2x Kapa Hifi Master Mix, 10 μM 10x Genomics SI-PCR primer, and 10 μM Nextera indexing primer (CAAGCAGAAGACGGCATACGAGATxxxxxxxxGTCTCGTGGGCTCGGAGATGTGTATAAGAGACAG).

##### Sequencing

For 3P libraries, the samples were pooled in a ratio of 80% RNA, 12% ADT, and 8% HTO.

For the 5P libraries, the samples were pooled in a ratio of 70% RNA, 12% ADT, 8% HTO, 5% of TCR libraries (with equal amounts of α/β and γ/δ libraries), and 5% of BCR libraries. 3P and 5P libraries were then pooled together in equal amounts and sequenced on an Illumina Novaseq S4 flowcell.

#### Validation of targeted immunophenotype panels experiments

Commercially available cryopreserved PBMCs (AllCells) were thawed into DMEM with 10% FBS. Two million cells per condition (4 conditions) were spun down in Eppendorf tubes at 4°C for 5 min at 400 *g*, and resuspended in 100 μl PBS with 2% BSA. Each aliquot was incubated for 10 min with 10 μL of FcX block, followed by staining with flow cytometry antibodies (BioLegend) on ice for 30 min. Cells were washed three times with PBS with 2% BSA. Samples were then gated as described below and sorted directly into Buffer RLT (QIAGEN).

#### Antibodies used (all at 5uL per condition unless otherwise noted):

**Table:** 

Specificity	Fluorophore	Clone	Note
CD3	AF488	UCHT1	10*u*L of antibody used
CD8	APC-Cy7	SK1	
CD4	AF700	RPA-T4	
CD57	PE	QA17A04	
CD56	APC	5.1H11	
CD103	BV421	Ber-ACT8	
Integrin 7	PE	FIB504	
CD49a	APC	TS2/7	BioLegend
CD43	PE	CD43-10G7	BioLegend

Gating conditions for each of the validation experiments are shown in Figures 4D and 4E.

Post sorting, samples were each split into quintuplicates, and then cleaned up with 2x SPRI. Samples were then brought into reverse transcription in an adaptation of SMARTseq2 (Picelli et al., 2014) and SCRB-seq (Soumillon et al., 2014) as described here: https://dx.doi.org/10.17504/protocols.io.nkgdctw.

The pooled library was sequenced on an Illumina Nextseq (50 R1, 8 index, 34 R2). Post base calling, samples were aligned using a wrapper for DropSeqTools against the human reference hg19 to generate RNA counts matrices.

To assess the agreement between single-cell datasets and bulk-sorted experiments, we examined the top DE genes separating our gated populations in the CITE-seq reference dataset. We next visualized the relative expression of these genes in the heatmaps in Figures 4D and 4E. The bulk-sorted populations exhibited highly concordant relative expression patterns for DE genes as we observed in CITE-seq data.

#### Flow cytometry analysis of whole blood

Whole blood collected immediately before the first VSV *gag* administration and then 1 and 7 days after was stained in TruCOUNT tubes as previously described (Elizaga et al., 2018; Hensley et al., 2012; Hensley-McBain et al., 2014) using the following antibody staining panel: (antibodies from BD Biosciences, unless otherwise indicated): CD14–V450, CD19–V450, CD45–AmCyan, CD4–FITC, CD8–PerCP-Cy5.5, CD123–PE, HLA-DR–ECD (Beckman Coulter), CD86–PE-Cy5, CD56–PE-Cy7, CD11c–APC, CD3–Alexa700 and CD16–APC-Cy7. We used these measurements in Figures 6G and 6H to validate changes in cell type abundance that were detected by scRNA-seq.

#### Determination of cellular responses to CMV

Intracellular cytokine staining assays were conducted as described in Li et al. (2017) and the proportion of CD8^+^ T cells expressing IL-2 and/or IFN-γ after stimulation with a CMV peptide pool as well as the response call are listed are in Table S1.

#### Mass cytometry

PBMC from patients with nasopharyngeal swab PCR-confirmed COVID-19 and healthy controls were thawed into warm RPMI (HyClone/Thermo Scientific) supplemented with 10% FBS and 0.5 × 106 cells per sample were transferred into a 96-well plate for staining. Cells were stained as previously described in Vendrame et al. (2020), using the panel described in McKechnie et al. (2020) with the addition of the following antibodies to identify MAIT cells: anti-CD161 (DX12, BD Biosciences) conjugated on 141Pr and TCR Vα7.2 (clone 3C10, Biolegend) conjugated on 162Dy. Antibodies were conjugated using MaxPar® X8 Conjugation Kits (Fluidigm, South San Francisco, CA, USA) or purchased pre-conjugated from Fluidigm. With the exception of the antibodies added, the immune profiling panel was premixed and frozen at −80°C in order to ensure antibody stability and minimize differential staining between batches as described in McKechnie et al. (2020). Briefly, cells were washed with PBS (Rockwell) and resuspended in 25 mM cisplatin (Enzo, Farmingdale, NY, USA) for sixty seconds to stain for viability before being quenched with undiluted FBS. Samples were multiplexed by staining with CD45-Pd barcodes as previously described (Mei et al., 2015), washed thoroughly in CyFACS buffer (PBS, 0.1% BSA, 2mM EDTA, 0.05% sodium azide), and pooled into sets of barcodes. Barcoded samples were then stained with all antibodies for 30 min, washed with CyFACS buffer, and fixed in 2% Paraformaldehyde (Electron Microscopy Sciences, Hatfield, PA, USA) for 20 min at room temperature. Fixed cells were permeabilized with 1x eBiosciences Permeabilization Buffer. Manufacturer ThermoFisher Scientific (Waltham, MA). Samples were washed, resuspended in 2% PFA containing iridium intercalator (Fluidigm), and stored at 4°C until acquisition (within 3 days of staining). On the day of acquisition, samples were washed once with PBS and thrice with Milli-Q water before being resuspended in 1 × EQ Beads (Fluidigm) and collected on a Helios mass cytometer (Fluidigm).

Prior to analysis, fcs files were debarcoded and bead-normalized with EQ beads using the Premessa package in the open-source statistical software R as previously described (Finck et al., 2013). FlowJo v10.7.1 was used to visualize the data and used to gate out beads, dead cells, doublets, and cell debris. MAIT cells were identified by expression of CD3, CD161, and TCR Vα 7.2 as shown in Figure S7.

#### Weighted Nearest Neighbor Analysis

The weighted nearest neighbor (WNN) procedure implemented in Seurat v4 is designed to integrate multiple types of data that are collected in the same cells to define a single unified representation of single-cell multimodal data. For each cell, the procedure learns a set of modality weights, which reflect the relative information content for each data type in that cell. This enables the generation of a WNN graph: for each cell, this graph denotes the most similar cells in the dataset based on a weighted combination of protein and RNA similarities. The WNN graph can be used as input for common downstream analytical tasks including tSNE or UMAP visualization, graph-based clustering, and the identification of developmental trajectories.

Our approach consists of four broad steps, as explained in detail below: (1) Constructing independent *k*-nearest neighbor (KNN) graphs for both modalities. (2) Performing within and across-modality prediction (3) Calculating cell-specific modality weights. (4) Calculating a WNN graph.

All methods are implemented in our open-source R package Seurat (https://www.satijalab.org/seurat, https;//www.github.com/satijalab/seurat).

##### Constructing k-nearest neighbor graphs for each modality

The WNN procedure begins by first applying standard analytical workflows to each modality independently and constructing KNN graphs for each one. In this manuscript we analyze data falling into three categories: measurements of single-cell gene expression, single-cell surface protein expression, and single-cell chromatin accessibility (ATAC-seq). For most analyses in this manuscript, we use a default value of *k* = 20, which is also the default value of *k* in the standard Seurat clustering workflow. For the analysis of the multimodal PBMC atlas, due to the substantial size of the dataset, we used a value of *k* = 30. In Figure S2, we show that we obtain very similar results from the WNN procedure when varying *k* across a series of values ranging from 10 to 50.

For clarity, we overview the analytical workflows for each data type below:

Single-cell gene expression: We analyze scRNA-seq data using standard pipelines in Seurat which include normalization, feature selection, and dimensional reduction with PCA. We then construct a KNN graph after dimensional reduction.

We emphasize that WNN analysis can leverage any scRNA-seq preprocessing workflow that generates a KNN graph. For example, users can preprocess their scRNA-seq data with a variety of normalization tools including log-normalization, scran (Lun et al., 2016) or SCTransform (Hafemeister and Satija, 2019), and can utilize alternative dimensional reduction procedures such as factor analysis or variational autoencoders. In this manuscript, we use workflows that are available in the Seurat package, and detail exact settings for each analysis later in this document.

Single-cell cell surface protein level expression: We analyze single-cell protein data (representing the quantification of antibody-derived tags (ADTs) in CITE-seq or ASAP-seq data) using a similar workflow to scRNA-seq. We normalize protein expression levels within a cell using the centered-log ratio (CLR) transform, followed by dimensional reduction with PCA, and subsequently construct a KNN graph. Unless otherwise specified, we do not perform feature selection on protein data, and use all measured proteins during dimensional reduction.
Single-cell chromatin accessibility: We analyze single-cell ATAC-seq data using our previously described workflow (Stuart et al., 2019), as implemented in the Signac package. We reduced the dimensionality of the scATAC-seq data by performing latent semantic indexing (LSI) on the scATAC-seq peak matrix, as suggested by Cusanovich et al. (2018). We first computed the term frequency-inverse document frequency (TF-IDF) of the peak matrix by dividing the accessibility of each peak in each cell by the total accessibility in the cell (the “term frequency”), and multiplied this by the inverse accessibility of the peak in the cell population. This step ‘upweights’ the contribution of highly variable peaks and down-weights peaks that are accessible in all cells. We then multiplied these values by 10,000 and log-transformed this TF-IDF matrix, adding a pseudocount of 1 to avoid computing the log of 0. We decomposed the TF-IDF matrix via SVD to return LSI components, and scaled LSI loadings for each LSI component to mean 0 and standard deviation 1.

As described for scRNA-seq analysis, while we use Seurat and Signac functions in this manuscript, any analytical workflow that computes a KNN graph for surface protein or chromatin accessibility data can also be used in the first step of WNN analysis.

##### Performing within and cross-modality predictions

Suppose we have a CITE-seq dataset where two modalities, RNA and protein, are measured in each single cell. From the previous step, we define the following:

$r_{i}$: L2-normalized low-dimensional vector representing the RNA profile for cell i
$p_{i}$: L2-normalized low-dimensional vector representing the protein profile for cell i
$\left\{knn_{r,i,1}\ldots knn_{r,i,k}\right\}$: the set of k-nearest RNA neighbors for cell i
$\left\{knn_{p,i,1}\ldots knn_{p,i,k}\right\}$: the set of k-nearest protein neighbors for cell i

We average the low-dimensional profiles of each neighbor set, which represent a prediction for the molecular contents for cell i based on their local neighborhoods. We perform both within-modality and cross-modality prediction:

Within-modality prediction:

$${\hat{r}}_{i,knn_{r}}=\frac{\sum_{j=1}^{k}r_{knn_{r,i,j}}}{k}\;\text{:}\;\text{prediction of RNA profile for cell}\;i\text{,}\;\text{based on RNA neighbors}$$

$${\hat{p}}_{i,knn_{p}}=\frac{\sum_{j=1}^{k}p_{knn_{p,i,j}}}{k}\;\text{:}\;\text{prediction of protein profile for cell}\;i\text{,}\;\text{based on protein neighbors}$$

Cross-modality prediction:

$${\hat{r}}_{i,knn_{p}}=\frac{\sum_{j=1}^{k}r_{knn_{p,i,j}}}{k}\text{:}\;\text{prediction of RNA profile for cell}\;i\text{,}\;\text{based on protein neighbors}$$

$${\hat{p}}_{i,knn_{r}}=\frac{\sum_{j=1}^{k}p_{knn_{r,i,j}}}{k}\text{:}\;\text{prediction of protein profile for cell}\;i\text{,}\;\text{based on RNA neighbors}$$

##### Calculating cell-specific modality weights

We next calculate the similarity between predicted values for each cell ${\hat{r}}_{i}$ and ${\hat{p}}_{i}$, and the actual values $r_{i}$ and $p_{i}$. We first compute Euclidean distances between predicted and actual values, and next convert these to affinities using the exponential kernel utilized in UMAP (McInnes et al., 2018). In McInnes et al. (2018), the authors demonstrate that the distance between a cell and its first nearest neighbor (‘local connectivity’) functions as distance gap that inflates values in both the numerator and denominator of the exponent. Therefore, they subtract local connectivity from cellular distances when computing the exponential kernel.

$$\theta_{rna}\left(r_{i},{\hat{r}}_{i,knn_{r}}\right)=\exp\left(\frac{-\max\left(d\left(r_{i},{\hat{r}}_{i,knn_{r}}\right)-d\left(r_{i},r_{knn_{r,i,1}}\right),0\right)}{\sigma_{r,i}-d\left(r_{i},r_{knn_{r,i,1}}\right)}\right)\;\text{affinity between}\;r_{i}\;\text{and predicted RNA profile }\left(\text{based on RNA knn}\right)$$

$$\theta_{rna}\left(r_{i},{\hat{r}}_{i,knn_{p}}\right)=\exp\left(\frac{-\max\left(d\left(r_{i},{\hat{r}}_{i,knn_{p}}\right)-d\left(r_{i},r_{knn_{r,i,1}}\right),0\right)}{\sigma_{r,i}-d\left(r_{i},r_{knn_{r,i,1}}\right)}\right)\;\text{affinity between}\;r_{i}\;\text{and predicted RNA profile }\left(\text{based on protein knn}\right)$$

$$\theta_{protein}\left(p_{i},{\hat{p}}_{i,knn_{p}}\right)=\exp\left(\frac{-\max\left(d\left(p_{i},{\hat{p}}_{i,knn_{p}}\right)-d\left(p_{i},p_{knn_{p,i,1}}\right),0\right)}{\sigma_{p,i}-d\left(p_{i},p_{knn_{p,i,1}}\right)}\right)\;\text{affinity between}\;p_{i}\;\text{and predicted protein profile }\left(\text{based on protein knn}\right)$$

$$\theta_{protein}\left(p_{i},{\hat{p}}_{i,knn_{r}}\right)=\exp\left(\frac{-\max\left(d\left(p_{i},{\hat{p}}_{i,knn_{r}}\right)-d\left(p_{i},p_{knn_{p,i,1}}\right),0\right)}{\sigma_{p,i}-d\left(p_{i},p_{knn_{p,i,1}}\right)}\right)\;\text{affinity between}\;p_{i}\;\text{and predicted protein profile }\left(\text{based on RNA knn}\right)$$

In the equations above, *d* represents the Euclidean distance metric, and $\sigma_{r,i}$ and $\sigma_{p,i}$ represent the bandwidth of the RNA and protein kernels for cell i. A commonly used approach is to set the bandwidth of a kernel to reflect the distance between a cell and its *k-*th nearest neighbor, resulting in an adaptive bandwidth that is specific to each cell (Haghverdi et al., 2016; van Dijk et al., 2018). However, the value of *k* used to compute this bandwidth is typically fixed across all cells. We considered that cells originating from rare states should not have the same bandwidth constraint as cells originating from abundant states, and therefore considered a modified approach to select kernel bandwidths.

Our approach is inspired by the concept of large margin nearest neighbors, which aims to identify kernel bandwidths that separate data points in the same class from those in different classes, even if the classes are closely related (Weinberger and Saul, 2009). In the context of unsupervised single-cell analysis (where the data points are unlabeled), we aim to identify a kernel bandwidth that groups together cells in the same state, yet divides cells that originate from closely related (but different) states.

Recent work has clearly demonstrated that KNN-graphs are prone to the formation of spurious edges, which represent links between cells that share some similarity molecular profiles, but are not in a matched molecular state (Levine et al., 2015). However, it is possible to identify these spurious edges through the use of the Jaccard metric. This identifies the number of shared nearest neighbors between two cells, thereby exploiting the local density of each data point to separate well-supported from spurious edges.

For each cell i, we therefore aim to identify the 20 cells in the dataset with the *lowest* non-zero Jaccard similarity. We expect that these represent cells that exhibit some similarity with cell i, but are unlikely to reside in the same molecular state. If more than 20 cells share the same Jaccard value, we select the 20 with the furthest euclidean distance to cell i. We take the average of the Euclidean distances from cell i to the 20 selected cells, and set this as the cell-specific kernel bandwidth.

We next calculate the ratio between the affinities for $r_{i}$ with predictions based on RNA neighbors, and predictions based on protein neighbors. A large ratio suggests that the local neighborhood of the cell, as defined by its RNA neighbors, better reflects its molecular state. We calculate the analogous ratio for protein affinities. In both cases, we add a small ε(10^−4^) to the denominator to avoid numerical errors.

$$s_{rna}\left(i\right)=\frac{\theta_{rna}\left(r_{i},{\hat{r}}_{i,knn_{r}}\right)}{\theta_{rna}\left(r_{i},{\hat{r}}_{i,knn_{p}}\right)+\varepsilon },\;s_{protein}\left(i\right)=\frac{\theta_{protein}\left(p_{i},{\hat{p}}_{i,knn_{p}}\right)}{\theta_{protein}\left(p_{i},{\hat{p}}_{i,knn_{r}}\right)+\varepsilon }$$

Finally, we normalize these values with a softmax transformation. The resulting two values are non-negative, and together sum to 1. We refer to these as cell-specific modality weights.

$$w_{rna}\left(i\right)=\frac{e^{s_{rna}\left(i\right)}}{e^{s_{rna}\left(i\right)}+e^{s_{protein}\left(i\right)}},\;w_{protein}\left(i\right)=\frac{e^{s_{protein}\left(i\right)}}{e^{s_{rna}\left(i\right)}+e^{s_{protein}\left(i\right)}}$$

##### Calculating a WNN graph

We leverage the cell-specific modality weights calculated above to define a new similarity metric between any two cells, which reflects a weighted combination of RNA and protein affinities. For two cells i and cell j, we define their weighted similarity as:

$$\theta_{weighted}\left(i,j\right)=w_{rna}\left(i\right)\theta_{rna}\left(r_{i},r_{j}\right)+w_{protein}\left(i\right)\theta_{protein}\left(p_{i},p_{j}\right)$$

We then construct a WNN graph, defined as a KNN graph constructed using this weighted similarity metric. For each cell, we consider the set

$$knn_{r,i,1}\ldots knn_{r,i,200}\cup knn_{p,i,1}\ldots knn_{p,i,200}\;\text{ and identify the k}-\text{most similar cells within this set based on the weighted similarity metric as weighted nearest neighbors}$$

##### Extending WNN to process more than two simultaneously measured modalities

The WNN method can be generalized to analyze single-cell datasets with three or more simultaneously measured modalities. Briefly, we perform within-modality comparisons for each modality, and extend the concept of cross-modality predictions to all pairwise combinations of modalities. We calculate affinity rations comparing within-modality predictions to cross-modality predictions and normalize these values with a softmax transformation. These ideas are a generalization of the methods described for two modalities, with a full mathematical description below for clarity:

Suppose the single-cell dataset has *M* modalities, we define the following:

*m, n*: Two different modalities in the dataset m∈[1,2,…M],n∈[1,2,…M],m≠n,
$X_{i}^{m}$: L2-normalized low-dimensional vector representing the modality *m* profile for cell i
$\left\{knn_{m,i,1}\ldots knn_{m,i,k}\right\}$: the set of k-nearest neighbors from modality *m* for cell i

Within-modality prediction:

$${\hat{X}}_{i,knn_{m}}^{m}=\frac{\sum_{j=1}^{k}X_{knn_{m,i,j}}^{m}}{k}\text{:}\;\text{prediction of }{\text{X}}_{i}^{m}\text{, based on neighbors from modality }m$$

Pairwise cross-modality prediction:

$${\hat{X}}_{i,knn_{n}}^{m}=\frac{\sum_{j=1}^{k}X_{knn_{n,i,j}}^{m}}{k},m\neq n\text{:}\;\text{prediction of}\;X_{i}^{n}\text{, based on neighbors from modality }m$$

We next calculate the within and cross-modality affinities, $\theta_{m,m}\left(X_{i}^{m},{\hat{X}}_{i,knn_{m}}^{m}\right)$ and $\theta_{m,n}\left(X_{i}^{m},{\hat{X}}_{i,knn_{n}}^{m}\right)$ between within and cross-modality predicted values for each cell ${\hat{X}}_{i,knn_{m}}^{m}$ and ${\hat{X}}_{i,knn_{n}}^{m}$, and the actual values $X_{i}^{m}$.

Within-modality affinity:

$$\theta_{m,m}\left(X_{i}^{m},{\hat{X}}_{i,knn_{m}}^{m}\right)=\exp\left(\frac{-\max\left(d\left(X_{i}^{m},{\hat{X}}_{i,knn_{m}}^{m}\right)-d\left(X_{i}^{m},X_{knn_{m,i,1}}^{m}\right),0\right)}{\sigma_{m,i}-d\left(X_{i}^{m},X_{knn_{m,i,1}}^{m}\right)}\right)$$

Pairwise cross-modality affinity:

$$\theta_{m,n}\left(X_{i}^{m},{\hat{X}}_{i,knn_{n}}^{m}\right)=\exp\left(\frac{-\max\left(d\left(X_{i}^{m},{\hat{X}}_{i,knn_{n}}^{m}\right)-d\left(X_{i}^{m},X_{knn_{m,i,1}}^{m}\right),0\right)}{\sigma_{r,i}-d\left(X_{i}^{m},X_{knn_{m,i,1}}^{m}\right)}\right),m\neq n$$

Pairwise affinity ratios (we add a small ε(10^−4^) to the denominator to avoid numerical errors):

$$s_{m,n}\left(i\right)=\frac{\theta_{m,m}\left(X_{i}^{m},{\hat{X}}_{i,knn_{m}}^{m}\right)}{\theta_{m,n}\left(X_{i}^{m},{\hat{X}}_{i,knn_{n}}^{m}\right)+\varepsilon },m\neq n$$

Finally, we normalize these pairwise affinity ratios with a softmax transformation. The resulting *m* modality weights for each cell are non-negative and together sum to 1.

$$w_{m}\left(i\right)=\frac{\sum_{n}e^{s_{m,n}\left(i\right)}}{\sum_{m}\sum_{n}e^{s_{m,n}\left(i\right)}},m\neq n$$

For two cells i and cell j, we define their weighted similarity as:

$$\theta_{weighted}\left(i,j\right)=\sum_{m}w_{m}\left(i\right)\theta_{m}\left(i,j\right)$$

We then construct a WNN graph, defined as a KNN graph constructed using this weighted similarity metric. For each cell, we consider the set $knn_{1,i,1}\ldots knn_{1,i,200}\cup knn_{2,i,1}\ldots knn_{2,i,200}\cup \ldots \cup knn_{M,i,1}\ldots knn_{M,i,200}$ and identify the *k*-most similar cells within this set based on the weighted similarity metric as weighted nearest neighbors.

#### Preprocessing details for each dataset

##### Cord blood mononuclear cells (CBMC) CITE-seq dataset

This CBMC dataset is a CITE-seq dataset from Stoeckius et al. (2017) and contains 8,617 cells with a panel of ten antibodies. We use the expression matrices as quantified in the original experiment. This experiment includes a small proportion of spiked-in murine 3T3 cells as negative controls. We apply SCTransform (Hafemeister and Satija, 2019) to normalize gene expression data, and we apply a CLR transformation to normalize protein data within each cell. We use PCA to reduce the dimensionality of both datasets, taking 30 RNA and 7 protein dimensions to construct the WNN graph.

##### Human bone marrow mononuclear cells (BMNC) CITE-seq dataset

The BMNC dataset is a CITE-seq dataset from Stuart et al. (2019), consisting of 30,672 cells with a panel of 25 antibodies. We use the expression matrices as quantified in the original experiment. For gene expression, in order to facilitate comparisons with other methods, we use standard log-normalization with default parameters in Seurat. We apply a CLR transformation to normalize protein data within each cell. We use PCA to reduce the dimensionality of both datasets, taking 30 RNA and 18 protein dimensions to construct the WNN graph. When performing a targeted re-clustering of T cell populations (Figure S2I), we repeated all preprocessing steps and performed the same procedure on 14,901 cells identified as T cells.

##### ASAP-seq dataset of human PBMC

We used the published human PBMC ASAP-seq dataset from Mimitou et al. (2020), containing 4,725 cells with a panel of 227 antibodies. We use the ADT expression matrix, ATAC fragment files, and QC parameters from the original publication. We called peaks from the ATAC fragment files using the MACS2 callpeak function (Zhang et al., 2008), and kept all peaks with -LOG10(qvalue) > 5 for the downstream ATAC analysis. We apply TFIDF to normalize ATAC peaks and CLR transformation to normalize protein data within each cell. We use LSI to reduce the dimensionality of ATAC normalized data, and PCA to reduce the dimensionality of protein. Then, we used LSI dimensions 2-50 LSI dimensions (excluding the first dimension as this is typically correlated with technical metrics in ATAC-seq data), and 30 protein PCA dimensions to construct the WNN graph.

##### 10x multiome ATAC+Gene expression dataset of human PBMC

10x Genomics multiomic (RNA + ATAC) data for human PBMCs was obtained from 10X website (https://support.10xgenomics.com/single-cell-multiome-atac-gex/datasets) and was processed using Signac (Stuart et al., 2020) and Seurat. ATAC-seq peaks were then identified for each cell type separately using MACS2, using the function CallPeaks in Signac 1.1.0 with arguments group.by = ‘celltype’ and additional.args = ‘–max-gap 50’. Fragment counts for each peak were quantified per cell using the FeatureMatrix function in Signac. Per-cell quality control metrics were computed using the TSSEnrichment and NucleosomeSignal functions, and cells retained with a nucleosome signal score < 2, TSS enrichment score > 1, and total RNA counts < 100,000 and > 25,000. We apply SCTransform to normalize RNA counts and TFIDF to normalize ATAC peaks. We use LSI to reduce the dimensionality of ATAC data, and PCA to reduce the dimensionality of RNA. Then, we used 2-40 LSI dimensions and 1-40 RNA PCA dimensions to construct the WNN graph.

##### SHARE-seq ATAC+Gene expression dataset of mouse skin cells

SHARE-seq data for mouse skin cells was obtained from GEO: GSE140203 (Ma et al., 2020), and was processed using Signac and Seurat. We used the ATAC-seq peak calls, peak by cell quantifications, and fragment files from the original publication. We applied SCTransform to normalize RNA counts and TFIDF to normalize ATAC peaks. We used LSI to reduce the dimensionality of ATAC data, and PCA to reduce the dimensionality of RNA. Then, we used LSI dimensions 2-30 (excluding the first dimension as this is typically correlated with technical metrics in ATAC-seq data) and 30 RNA PCA dimensions to construct the WNN graph. Cells are annotated by their original annotations in the Ma et al. (2020) except for four basal subpopulations which are annotated by WNN-derived clusters. Motif analyses for the ASAP-seq, 10x RNA^+^ATAC and SHARE-seq datasets followed the suggested workflow described at https://satijalab.org/signac/articles/motif_vignette.html

##### PBMC CITE-seq datasets of HIV Vaccine Trials Network samples

Alignment and expression quantification: We applied standard pipelines to initially align and quantify the CITE-seq datasets newly generated for this manuscript. For both the 10x v3 (3′ scRNaseq) and 10x Immune Profiling Solution (5′ scRNA-seq), we used Cell Ranger 3.1.0 to align reads to the GRCh38 human genome with default settings. To quantify libraries of hashtag oligos (HTO) from cell hashing, or antibody-derived tags (ADT) from CITE-seq, we used Alevin (Srivastava et al., 2019). A dictionary of barcode sequences for each antibody clone is included in Table S2.

Quality control and doublet removal: We considered all cells that were detected in our RNA-seq, cell hashing, and ADT libraries. We first filtered out cells with that were outliers for the number of detected features from these modalities. We removed cells with < 500 detected genes, but also removed cells where we detected an aberrantly high number of features (more than 6,000 genes, more than 50,000 ADT reads, or more than 10,000 ADT reads), particularly to avoid clumps of antibodies that can occasionally attach to cells. We used our previously described hashing-based doublet detection strategy (Stoeckius et al., 2018), implemented in HTODemux, to identify doublets that represent two or more cells representing different samples. Inspired by the scrublet package (Wolock et al., 2019), we implemented a strategy to further remove doublets that may originate within the same sample (and would therefore not be identified through cell hashing). We first constructed a KNN graph based on the ADT data. For each cell, we examined the percentage of neighbors that had been marked by HTODemux as doublets. If this percentage exceeded 20%, we reasoned that the cell’s molecular profile was similar to a verified doublet, and therefore removed it from further analysis.

Sample integration (10X 3′ CITE-seq experiments): To facilitate the identification of shared cell types across datasets, we applied our previously developed ‘anchor’ workflow (Stuart et al., 2019) to integrate the datasets. We partitioned the dataset into 24 groups, each corresponding to one of the original samples representing one of eight volunteers, and one of three time points. To integrate the gene expression values, we first separately normalized each of the 24 groups using SCTransform, and applied the reciprocal PCA workflow, which is optimized for integration tasks with large numbers of samples and cells. When performing integration, we designated the unvaccinated cells (day 0), as reference datasets. We integrated the protein measurements across samples using the same workflow, but after performing normalization within each cell using a CLR transformation.

We reduce the dimensionality of the integrated gene expression and integrated protein datasets via PCA. We use the top 40 and 50 dimensions respectively to construct KNN graphs from the RNA and protein modalities, which is used as input to the WNN procedure described above.

Clustering and annotation: To cluster our multimodal dataset, we first used the KNN graph based on the weighted RNA and protein similarities (referred to as the WNN graph), to calculate the Jaccard index (neighborhood overlap) between every pair of cells. This distance represents the edge weight in a shared nearest neighbor graph (SNN), which we used as input to the graph-based smart local moving (SLM) algorithm (Waltman and Van Eck, 2013). We initially clustered cells at a high resolution (resolution = 5), and performed differential expression (see below) on all pairs of clusters for both RNA and protein markers. We merged clusters that did not exhibit clear evidence of separation, or where the only differentially expressed features represented ribosomal genes or mitochondrial genes. In some cases (particularly for extremely rare cell types that required a higher resolution to be correctly annotated in our clustering), we increased the granularity of our clustering by subsetting cells in an individual cluster, and rerunning SLM on this subgraph. In our final annotations, we considered 57 total clusters.

We note that the annotation process requires careful consideration of both known RNA and protein markers, as well as those that are discovered through unsupervised analysis. We placed clusters into eight broad groups (Level 1 annotations: CD4^+^ T cells, CD8^+^ T cells, Unconventional T, B cells, Natural Killer (NK) cells, Monocytes, Dendritic Cells (DC), and Other (consisting of progenitors and additional rare populations expressing erythroid or platelet lineage markers). We further subdivided these groups into 30 Level 2 annotation categories representing well-described subtypes of human immune cells: CD4^+^ T Naive, CD4^+^ T Central Memory (TCM), CD4^+^ T Effector Memory (TEM), CD8^+^ TEM, etc., all thirty subtypes are listed at https://azimuth.hubmapconsortium.org/). Our 57 clusters fall into subsets of these categories (i.e., CD8^+^ TCM_1, CD8^+^ TCM_2, etc.), and represent Level 3 annotations with the highest level of granularity (all listed in the legend for Figure 3C). We report markers for each of our Level 3 annotations in Figure S4.

#### Simulated addition of protein noise

In Figure 2C, we perform a robustness analysis to explore the effects of artificially reducing the information content in one data type. To achieve this, we add increasing amounts of random noise to the protein data, immediately prior to running PCA. The amount of noise added is generated independently for each element in the matrix, and is drawn from a Gaussian distribution with mean zero and increasing standard deviation (sd = 0.5, 1, 1.5, 2, 3, 4, 5). After adding noise, we repeated the WNN procedure.

#### Comparing transcriptomic heterogeneity of WNN, RNA, and ADT-derived neighborhoods

In addition, we sought to ensure that the incorporation of protein information in the WNN graph does not come at the expense of identifying transcriptomically congruent neighborhoods. We therefore examined the WNN-derived neighborhoods for bone marrow cells originating from clusters with predominantly high protein weights, such as regulatory T cells, and compared them with RNA-derived neighborhoods. If the WNN procedure is performing well, gene expression levels within these neighborhoods should represent a ‘homogeneous’ population with low levels of variability. We therefore compared levels of gene variability between RNA-derived and WNN-derived neighborhoods.

We utilized two different measures to quantify the heterogeneity of gene expression within a local neighborhood (STAR Methods). Inspired by the M3Drop (Andrews and Hemberg, 2019), we identified ‘variable’ features in a group of cells based on unexpectedly high ‘dropout’ rates after controlling for a gene’s average expression. We found that WNN-derived neighborhoods were most reflective of a homogeneous population, as evidenced by the number of genes that exhibited variable levels of expression within each neighbor set (Figure S2). We obtained similar results when computing a measure of ‘excess variance’, defined as the amount of residual variance observed for each gene after controlling for the mean-variance relationship inherent in single-cell data, and also when repeating these analyses using progenitor subpopulations with high RNA modality weights (Figure S2). Moreover, we found that differentially expressed genes between cell states exhibited nearly identical fold-changes in either WNN-derived or RNA-derived clusters (Figure S2).

Specifically, in Figures S2J–S2M, we test whether the WNN graph generates local neighborhoods that exhibit congruent (or ‘homogeneous’) levels of gene expression. In particular, we sought to confirm this for subpopulations comprising cells with high protein modality weights, such as the 297 regulatory T cells in our BMNC dataset. We therefore considered three sets of 5,940 (297 ^∗^ k = 20) neighbor cells, defined using either the RNA, ADT, or WNN neighbor graphs, and attempted to identify ‘variable’ genes within these cells as a test of heterogeneity.

In Figure S2J, we plot the relationship between pseudobulk-level gene expression and ‘dropout’ rate, as inspired by M3Drop (Andrews and Hemberg, 2019). We fit an average trendline using the ksmooth function from package stats with a Gaussian kernel and default parameters, and calculate the residual of each gene to the fitted line. We consider genes with a residual > 0.1 to be variable. In Figure S2L we perform the same analysis, but utilize the standard deviation of gene expression across single cells as an alternative metric to ‘dropout’ rate for defining variable genes (residual from trendline > 0.5). We repeat the same analysis for HSC (defined by high RNA modality weights) in Figures S2K and S2M.

#### Differential analysis for clusters defined by RNA and WNN

In Figures S2N and S2O, we compare the results of differential expression after performing clustering on either the RNA-derived or WNN-derived nearest neighbor graphs for the BMNC dataset. We first cluster cells by the RNA or WNN nearest neighbor graphs respectively, and annotate clusters based on their molecular profiles. While some clusters (for example, regulatory T cells) were only identified in the WNN-derived clusters, we did identify shared populations across both cluster sets including: CD4 Naive T, CD4 Memory T, CD8 Naive T, B Naive, B Memory, HSC, and LMPP subgroups. For both the WNN-derived and RNA-derived cluster sets, we performed four transcriptome-based differential expression tests (HSC versus LMPP, CD8 Naive versus CD4 Naive, CD4 Memory versus CD4 Naive, Naive B versus Memory B) using the Wilcoxon test implemented in Seurat. For genes identified as differentially expressed (adjusted p value < 0.01) in either the WNN-derived or RNA-derived cluster sets, we compared the difference in observed magnitude of log2 fold changes (Figures S2N and S2O).

#### Comparisons with MOFA+ and totalVI

In order to assess the performance of our WNN method alongside other recently proposed multimodal integration tools, we compared the results of WNN, Total Variational Inference (totalVI version 0.6.7) (Gayoso et al., 2019) and Multi-omics factor analysis v2 (MOFA+ version 1.1) (Argelaguet et al., 2020), on the BMNC dataset. We followed the recommended settings and workflows for both methods, and further describe parameter choices below.

For totalVI, we use the RNA and ADT counts matrices as input. We use the subsample_genes function to select 4000 variable genes, and used 500 epochs for model training, as suggested in the totalVI tutorial (https:// scvi-tools.org/en/stable/tutorials/totalvi.html). All other parameters were set to default settings. We identified nearest neighbors, and performed UMAP visualization on the learned latent space.

For MOFA+, we used the same normalization method as Seurat to facilitate direct comparison. As recommended in the MOFA+ tutorial (https://raw.githack.com/bioFAM/MOFA2_tutorials/master/R_tutorials/10x_scRNA_scATAC.html), we used the z-scored data (‘scaled’ data) from the two assays as view1 and view2 for MOFA+. All other parameters were set to default or recommended settings. We identified nearest neighbors, and performed UMAP visualization based on the learned factors.

The UMAP plots in Figures S2A and S2B show the results of all three methods (we also include independent RNA and protein analyses in Seurat for comparison). The plots show that the methods generally reveal similar sets of cell types, but with important differences. For example, regulatory T cells, defined by CD25 expression, are only separated in the WNN UMAP. Figure S2B demonstrates that this is due to the fact that CD25^+^ cells only form a distinct cluster in WNN analysis.

In order to move beyond visualization and quantify the performance of each method, we averaged the CD25 expression level for the calculated multimodal neighbors of each cell, returning a vector of predicted values. We quantified the performance of the method using the correlation (Pearson; Figure 2D, Spearman; Figure S2), between predicted and measured values. For CD25, WNN analysis achieved the highest correlation, as cells that are CD25^+^ are correctly identified as neighbors with other cells that are CD25^+^ in the dataset. We repeated this analysis for all protein features, and found that, WNN analysis consistently achieved the highest correlation. We repeated the analysis for all transcriptomic features as well (Figure S2) and observed similar performance for all methods. We note that transcriptomic correlations were also much lower, likely due to the substantial technical noise inherent to scRNA-seq data.

#### TCR analysis

To generate clonotype information for the 10X 5′ samples, TCRαβ and TCRγδ fastq files were processed with cellranger vdj version 3.0.2 against the GRCh38 v2.0.0 reference as provided by 10x Genomics. Clonotype information was then manually added into Seurat as cell metadata, allowing us to explore the relationship between annotated cell type, molecular state, and TCR sequence. We obtained productive TCR ⍺/β sequences representing 16,060 distinct clones, where all cells within a clone share the exact same CDR3⍺ and CDR3β sequences.

#### Identifying targeted immunophenotype panels

For each of our 57 clusters, we aimed to identify a reduced set of antibodies that could enrich for cells in this molecular state. We utilized forward feature selection with balanced logistic regression to identify targeted surface protein markers for each cell type. This represents an iterative process where we successively add markers based on a greedy algorithm aiming to maximize the classification power of logistic regression. Prior to initializing the procedure, we randomly downsampled cells within abundant cell states to ensure that no cluster made up more than 5% of all cells in the dataset. We used the implementation for logistic regression with 5-fold cross validation in the boot R package (Canty and Ripley, 2020). We ran ten rounds of forward selection, allowing us to design panels of one-ten immunophenotypic markers for each cell type. To enhance the interpretability of these panels, we required the first five markers selected to be positive markers. These panels are reported in Table S3. We used each panel to enrich for our 57 clusters ‘*in silico*’, using logistic regression with a decision boundary of 0.5 to set our gates. We report the enrichment, precision, and recall for each panel in Table S3.

#### Gradient analysis for NK and B cells

In Figures 5G, 5H, and S5, we identify genes whose expression level is correlated with a cell’s position along a molecular gradient defined by a single protein. For example, in Figure 5G, we ordered cells along a gradient defined by CD16 protein expression. We then calculated Moran’s I, a spatial autocorrelation metric proposed to identify trajectory-dependent genes in Monocle3 (Cao et al., 2019), to identify correlated genes. We plot a representative subset of these features in Figure 5G. We generate these plots by ordering cells on the x axis based on their expression level for CD16 protein, and apply the ksmooth function from package stats with default bandwidth and parameters (R Development Core Team, 2013) to calculate smoothed gene expression levels across the trajectory. We utilize the same approach for trajectory analyses based on CD38 and CD27.

#### Supervised principal component analysis for multimodal data

Due to the inherent levels of noise in single-cell RNA-seq, techniques such as PCA are often used to reduce the dimensionality of the dataset. PCA identifies correlated modules of genes, whose heterogeneous expression represent the largest sources of variance in the dataset. PCA is an unsupervised dimensional reduction technique, and while the correlated gene modules may typically represent markers of heterogeneous cell states in the dataset, they may also represent unwanted sources of variation related to technical noise, cell cycle state, or random fluctuations.

We therefore considered the application of supervised principal component analysis (sPCA) to our multimodal dataset. sPCA is a generalization of PCA that can be used not only for unsupervised learning, but also for regression and classification problems (Barshan et al., 2011). While PCA will identify the directions that explain maximal variance in the source data, sPCA can help pinpoint sources of variation that are of the greatest interest. To accomplish this, sPCA takes as input a kernel which describes the similarity between any two cells based on a response outcome. We set this kernel to represent the Jaccard distances derived from our WNN graph, as this considers the response outcome to be the weighted combination of RNA and protein profiles. sPCA will then estimate a set of principal components that have maximal dependence on the response variable (Barshan et al., 2011). These components should represent the optimal transcriptomic modules that can be used to separate the cell types defined in our multimodal dataset. Therefore, the sPCA procedure can identify the set of principal components that can transform the data in a single modality to best capture the structure in a multimodal dataset. We emphasize that sPCA takes as input a cell-cell similarity kernel, but does not require cells to be labeled or placed into discrete clusters. Therefore, it can capture both discrete and continuous sources of variation in a multimodal dataset.

Formally, sPCA transforms the dataset to maximize the dependency with the response variable. We implement the method described in Barshan et al. (2011), where the Hilbert-Schmidt Independence Criterion (HSIC) is used as the dependency measure. To apply this method in the context of single cell multimodal data, we define the following:

*X*: data matrix for gene expression measurements
*Y*: data matrix for protein measurements.
*U*: Transformation of *X* (for example, a set of principal components)
*K:* Kernel derived from *U*, describes the cell-cell similarity in *X*
*L*: Kernel derived from the WNN graph, and describes the cell-cell similarity based on a weighted combination of *X* and *Y*

The HSIC between two kernels *K* and *L* is:

$$HSIC\left(K,L\right)=\frac{1}{{\left(n-1\right)}^{2}}tr\left(KHLH\right)$$

The goal of the sPCA is to identify *U* that maximizes HSIC(K,L)

$$HSIC\left({\left(U^{T}X\right)}^{T}U^{T}X,L\right)=\frac{1}{{\left(n-1\right)}^{2}}tr\left(X^{T}UU^{T}XHLH\right)=\frac{1}{{\left(n-1\right)}^{2}}tr\left(U^{T}XHLHX^{T}U\right)$$

As described in described in Barshan et al. (2011), the optimization problem reduces to:

$$\begin{array}{l} \underset{U}{\text{argmax}}\;tr\left(U^{T}XHLHX^{T}U\right)\\ subject\;to\;U^{T}U=I \end{array}$$

where H is the centering matrix *H*_*ij*_ *= I – n*^*-1*^*ee*^*T*^.

This optimization problem has a closed form solution. *U* represents the eigenvectors of matrix *XHLHX*^*T*^, based on the top *d* eigenvalues, where *d* represents the desired number of components. Each vector in *U* describes the relative importance for each gene in defining this component (i.e, *U* represents a set of feature component loadings).

#### Mapping query datasets to a multimodal reference

We compute the sPCA transformation described above for our reference dataset, and can subsequently project this transformation onto any query dataset consisting of PBMC. This enables us to perform supervised analysis of the query datasets. Since our sPCA was computed based on a reference defined by a large number of cells and antibodies, this transformation will likely be more informative than an unsupervised PCA computed on a new scRNA-seq query. This transformation should therefore be more capable of separating cell types in the query dataset. As a secondary benefit, projecting the sPCA transformation onto a query dataset places the query in the same low-dimensional space as the reference. This provides a starting point to integrate the two datasets, which can assist in the visualization and annotation of the query as described below.

#### Reference-based Integration for query datasets

In Stuart et al. (2019), we demonstrate a workflow to identify reference-based transfer ‘anchors’ between reference and query datasets. Briefly, this workflow first projects a transformation calculated on the reference dataset onto the query. The method next identifies mutual nearest neighbors (Haghverdi et al., 2018) between the reference and query datasets, based on this L2-normalized low-dimensional space. These anchors can be used to transfer discrete or continuous data from the reference onto the query. For cell annotation (transfer of discrete label form reference to query), each query cell is assigned a label based on a weighted vote classifier, where each anchor provides a vote that is weighted by its similarity to the query cell. When classifying cells in this manuscript, we apply the same workflow, but use the sPCA transformation described above for projection.

In Stuart et al. (2019), we also provide methods to leverage an existing set of anchors in order to modify the underlying gene-expression levels, allowing shared cell types to cluster together across experiments. We apply a similar workflow here. However, instead of correcting values in high-dimensional space, we correct in low-dimensional space. This substantially improves the speed of the method. Moreover, at the conclusion of this procedure, we have placed the query dataset in the same low-dimensional representation (defined by the sPCA transformation) as the reference.

Having placed both the query and reference dataset in the same space, we have two options to visualize the query dataset. The first is that we can project the query data onto the same UMAP projection as has been previously computed. To accomplish this, we use the umap_transform functionality implemented in the R uwot package, which enables new points to be adding to an existing embedding. We use this approach to project query datasets onto the reference-defined visualization shown in Figure 3D. Together, these methods enable a fully automated pipeline to leverage a multimodal single-cell reference to annotate and visualize new single-cell query datasets, even if only the transcriptome was measured. To facilitate users applying this approach to interpret their own datasets from either healthy or diseased PBMC, we have provided a web application (https://azimuth.hubmapconsortium.org/) to automate these analyses.

A second option for visualization is to compute a new (‘de-novo’) UMAP visualization, which can be computed after merging the reference and query datasets together. For the analysis of the COVID-19 dataset (Wilk et al., 2020), we compute both visualizations. The reference-based UMAP is shown in Figure 7D, while the de-novo UMAP is shown in Figure S7. One advantage of the de-novo approach is that it can help to visualize populations in the query that cannot be effectively represented in the reference. For example, the Wilk et al. (2020) dataset contains subsets of neutrophils, activated granulocytes that were not captured in our reference, as well as subpopulations of monocytes whose expression profiles are heavily perturbed in COVID-19 samples. In the reference-based visualization, the umap_transform functions aims to embed these cells adjacent to their closest neighbors in the reference, which often places these cells at the boundary of cell clusters. In the de-novo visualization, all three of these populations remain distinct from reference cells even after integration (Figure S7). We encourage users to compute both to understand how their dataset can be interpreted in light of a reference, and also to flag any particular populations that may not be well represented.

We leverage this reference-based mapping workflow to interpret the 5′ scRNA-seq datasets generated for this manuscript. We use the same QC, normalization, and doublet filtration procedures to analyze the 5′ data as described earlier in this section. We apply the reference-based integrative analysis procedures described above to project the 5′ scRNA-seq data onto the UMAP visualization defined by the 3′ dataset, and also to transfer a discrete label. The annotation and projected UMAP are shown in Figure S5F, while the UMAP visualization with annotated clonotype structures is shown in Figure 5K.

Similarly, we applied the same pipeline to map the CITE-seq datasets from (Kotliarov et al., 2020). We downloaded the dataset at https://doi.org/10.35092/yhjc.c.4753772, applied SCTransform normalization, and repeated the mapping procedure applied above. While the dataset contains measurements for 82 proteins alongside the transcriptome, we used only the transcriptome for reference mapping and the transfer of Level 2 annotations. This allowed us to use the withheld protein data for benchmarking with scArches (version 0.1.2) (Lotfollahi et al., 2020), as shown in Figure S7.

#### Benchmarking Seurat reference-mapping with scArches

To run scArches, we followed the tutorial released by the authors. We first integrated our 24 3′ scRNA-seq samples into a reference atlas, using the same variable genes as used in the WNN analysis. We obtained poor results with the default nb loss function, and as suggested in the tutorial, tried the sse loss function as an alternative. We trained the scArches model using recommended parameter settings of 150 epochs and a batch size of 128, and next mapped query cells onto the reference using recommended parameters in the tutorial. To facilitate fair comparisons between our reference mapping workflow and scArches, we forced both methods to return the most likely annotation for each query cell.

We note the extensive challenges in benchmarking reference-based annotation workflows in the absence of ground-truth cell labels. By withholding the protein data from consideration during the mapping process, we can use the protein measurements as an independent assessment of prediction quality. For 35,619 cells (67.1%), Seurat and scArches returned the same annotation. For the remaining 17,480 query cells, the two methods returned two divergent annotations (for example, suppose that Seurat annotated the cell as CD4 Treg, and scArches annotated as NK). In the reference dataset, we calculated the protein centroids for the CD4 Treg and NK clusters. We then calculated the Pearson correlation between these centroids, and the protein values for the individual cell. If the cell’s protein levels exhibit a high correlation with the centroid of CD4 Treg, but a low correlation with the centroid of NK, this suggests that the Treg annotation is correct. This metric and approach are inspired by scmap (Kiselev et al., 2018). Essentially, in cases where two methods disagree based on an RNA classification, we attempt to classify the cell based on its protein levels to see if there is strong evidence for one annotation versus another. In 79.4% of cases, we observe stronger support for the Seurat annotation (Figure S7E).

### Quantification and statistical analysis

#### Differential abundance of cell types across experimental conditions

In Figures 6E and 6F, we analyze the composition of samples at different time points, and aim to find cell states whose abundance changes during the response. For Level 1 annotations, for each of the 24 samples, we calculated the percentage of each cell state in each sample, and ran two paired Wilcoxon tests: day 0 versus day 3, and day 0 versus day7. No cell states exhibited significant changes. To search for more subtle changes, we calculated the relative abundance of all 30 Level 2 annotations in each sample within each Level 1 group (for example, for each sample we calculated the fraction of CD14^+^ monocytes within the total pool of sample monocytes). These values were used as input to two paired Wilcoxon tests: day 0 versus day 3, and day 0 versus day7. We detected significant shifts (p < 0.05) for three clusters, visualized for the 10X 3′ samples in Figure 6F, with independent support for each of the signals in the 10X 5′ datasets (Figure S6).

#### Identifying differentially expressed genes across cell types and experimental time points

In this manuscript (for example, Figure 4A), we identify differentially expressed (DE) genes and proteins that represent biomarkers of different cell states, or represent specific responses across experimental conditions. We used the wilcoxauc method from presto (Korsunsky et al., 2019) to identify DE genes and proteins, reporting markers with adjusted p value < 10^−5^. For space considerations, we typically report only the top 20 markers in each heatmap, and sort genes first by adjusted p value and next by log fold-change to determine the top markers.

#### Perturbation score

In Figure 6A, we aim to identify the cell types whose molecular state exhibits significant changes during the response to vaccination. We note that when calculating DE genes and proteins within a cell state, across experimental time points, the statistical power of these per-gene tests is heavily dependent on the abundance of the cell state. We therefore considered an alternative metric, the ‘perturbation score’ as described in Papalexi et al. (2020), which quantifies the magnitude of the response across the transcriptome. To briefly summarize, we perform the following procedure to quantify the response for cells at day 3 versus day 0 for each cell state. We first identify a set of genes that exhibit initial evidence of differential expression across time points, but may not achieve statistical significance after multiple-testing correction (adjusted p value < 0.1). We compute the pseudobulk expression of these genes after grouping cells by experimental time point, generating a vector representing the average expression of these genes for day 0 cells, and a second vector representing the average expression at day 3 cells. We define the ‘perturbation vector’ for this cell state as the difference between these two vectors, normalized to length 1. Finally, we project the transcriptome of each cell onto this vector and quantify the magnitude of this projection. We find that this approach helps to prioritize cell types that exhibit robust responses, particularly when comparing populations with vastly different abundances.

#### Additional resources

Installation instructions, tutorials, and documentation for Seurat v4 are available at https://www.satijalab.org/seurat.

## Supplemental information

Table S1. Antibody information, related to Figure 3

Table S2. Panel enrichment, related to Figure 4

Table S3. Donor information, related to Figure 5
